# Preclinical models of hepatosplenic γδ T‐cell lymphoma with an activating STAT5B mutation display sensitivity to JAK inhibitor upadacitinib

**DOI:** 10.1002/hem3.70345

**Published:** 2026-04-16

**Authors:** Myint Myat Khine Aung, Susann Schönefeldt, Sophie Pfalz‐Kraupp, Tamara Wais, Tobias Suske, Safia Zahma, Stefan Franz, Andrea Müllebner, Gabriela Feurstein, Christina Wagner, Thomas Eder, Tea Pemovska, Christiane Agreiter, Alexander Pichler, Philipp B. Staber, Martin Hofer, Ralf Steinborn, Svenja‐Verena Strohmer, Ingrid Simonitsch‐Klupp, Marcus Bauer, Andreas Wilfer, Thomas Weber, Swapnil Potdar, Tero Aittokallio, Dennis Jungherz, Tony A. Müller, Julia List, Dagmar Gotthardt, Florian Grebien, Vasileios Bekiaris, Marco Herling, Richard Moriggl, Heidi A. Neubauer

**Affiliations:** ^1^ Centre for Biological Sciences University of Veterinary Medicine Vienna Vienna Austria; ^2^ Department of Internal Medicine I, Division of Hematology and Hemostaseology Medical University of Vienna Vienna Austria; ^3^ Department of Internal Medicine I (Oncology, Hematology, Clinical Immunology, and Rheumatology) Saarland University Medical School Homburg Germany; ^4^ VetCore Facility for Research/Infrastructure University of Veterinary Medicine Vienna Vienna Austria; ^5^ Genomics Core Facility, VetCore University of Veterinary Medicine Vienna Vienna Austria; ^6^ Department of Pathology Medical University of Vienna Vienna Austria; ^7^ Institute of Pathology Martin Luther University Halle‐Wittenberg Halle (Saale) Germany; ^8^ Department of Internal Medicine IV, Haematology and Oncology University Hospital Halle (Saale) Halle (Saale) Germany; ^9^ Institute for Molecular Medicine Finland (FIMM), HiLIFE University of Helsinki Helsinki Finland; ^10^ Institute for Cancer Research, Department of Cancer Genetics Oslo University Hospital Oslo Norway; ^11^ Oslo Centre for Biostatistics and Epidemiology (OCBE), Faculty of Medicine University of Oslo Oslo Norway; ^12^ Department of Hematology, Cell Therapy, Hemostaseology, and Infectious Diseases University of Leipzig Medical Center and Cancer Center Central Germany (CCCG) Leipzig‐Jena Leipzig Germany; ^13^ Department I of Internal Medicine University of Cologne Cologne Germany; ^14^ St. Anna Children's Cancer Research Institute (CCRI) Vienna Austria; ^15^ CeMM Research Center for Molecular Medicine of the Austrian Academy of Sciences Vienna Austria; ^16^ Department of Health Technology Technical University of Denmark Lyngby Denmark; ^17^ Department of Biosciences and Medical Biology Paris Lodron University Salzburg Salzburg Austria

Hepatosplenic T‐cell lymphoma (HSTCL) is a rare, aggressive mature T‐cell lymphoma that manifests predominantly in young adults with a median age of 34 years.^1^ HSTCL is typically characterized by the expansion of malignant γδ T cells causing hepatosplenomegaly with frequent involvement of the bone marrow (BM) and peripheral blood (PB). Approximately 20% of cases express an αβ T‐cell receptor (TCR), with αβ variants more frequently observed in females and patients over 50 years of age.^1^ HSTCL typically follows a rapidly progressive course and responds poorly to therapy. Preferred first‐line treatments for HSTCL patients include platinum/ifosfamide‐based induction regimens followed, when feasible, by an allogeneic hematopoietic stem cell transplantation.^2^ There are no approved targeted therapies, and prognosis remains poor, with a median overall survival of 11–59 months.^2^

Cytogenetic aberrations are common in HSTCL, with isochromosome 7q and trisomy 8 representing the most frequent chromosomal abnormalities.^1^, ^3^ Loss‐of‐function mutations are frequently reported in *SETD2*, *INO80*, *TET3*, and *SMARCA2*, and gain‐of‐function mutations often occur in *STAT5B*, *STAT3*, and *PIK3CD*.^3^, ^4^, ^5^ Notably, the oncogenic *STAT5B* ^*N642H*^ mutation is present in one‐third of patients and represents the most frequent mutation in HSTCL.^3^, ^6^ *STAT5B* ^*N642H*^ has been associated with inferior outcomes and increased relapse risk in patients with T‐cell malignancies,^7^, ^8^ suggesting that *STAT5B*‐mutant subclones may drive more aggressive, therapy‐resistant disease. We therefore established murine *STAT5B* ^*N642H*^‐driven γδ T‐cell lymphoma (γδTCL) cell lines and an allograft mouse model that recapitulate key features of HSTCL, and used these preclinical models together with patient‐derived HSTCL cells to evaluate JAK inhibitors (JAKi) as targeted therapeutics.

Murine γδ T cells expressing human STAT5B^N642H^ induce an aggressive γδTCL when transplanted from transgenic mice into wild‐type (WT) C57BL/6 mice.^9^ To generate a more robust, accessible system, we used ex vivo tumor cell outgrowth cultures from this model to establish three clonal, STAT5B^N642H^‐positive γδTCL cell lines (C2, C6, and C15) in the presence of IL‐2 (Figure 1A). STAT5B^N642H^ protein expression was verified by flow cytometry via the FLAG‐tag, and the transgene was detected by Sanger sequencing (Figure S1A,B). To assess HSTCL‐like features of the novel cell lines, we performed side‐by‐side comparisons with the two available human HSTCL cell lines, DERL‐2 and DERL‐7, established from a single HSTCL patient^10^ harboring the *STAT5B* ^*N642H*^ mutation.^11^, ^12^ Sanger sequencing revealed heterozygosity in DERL‐2 and homozygosity in DERL‐7 (Figure S1C).

**Figure 1:**
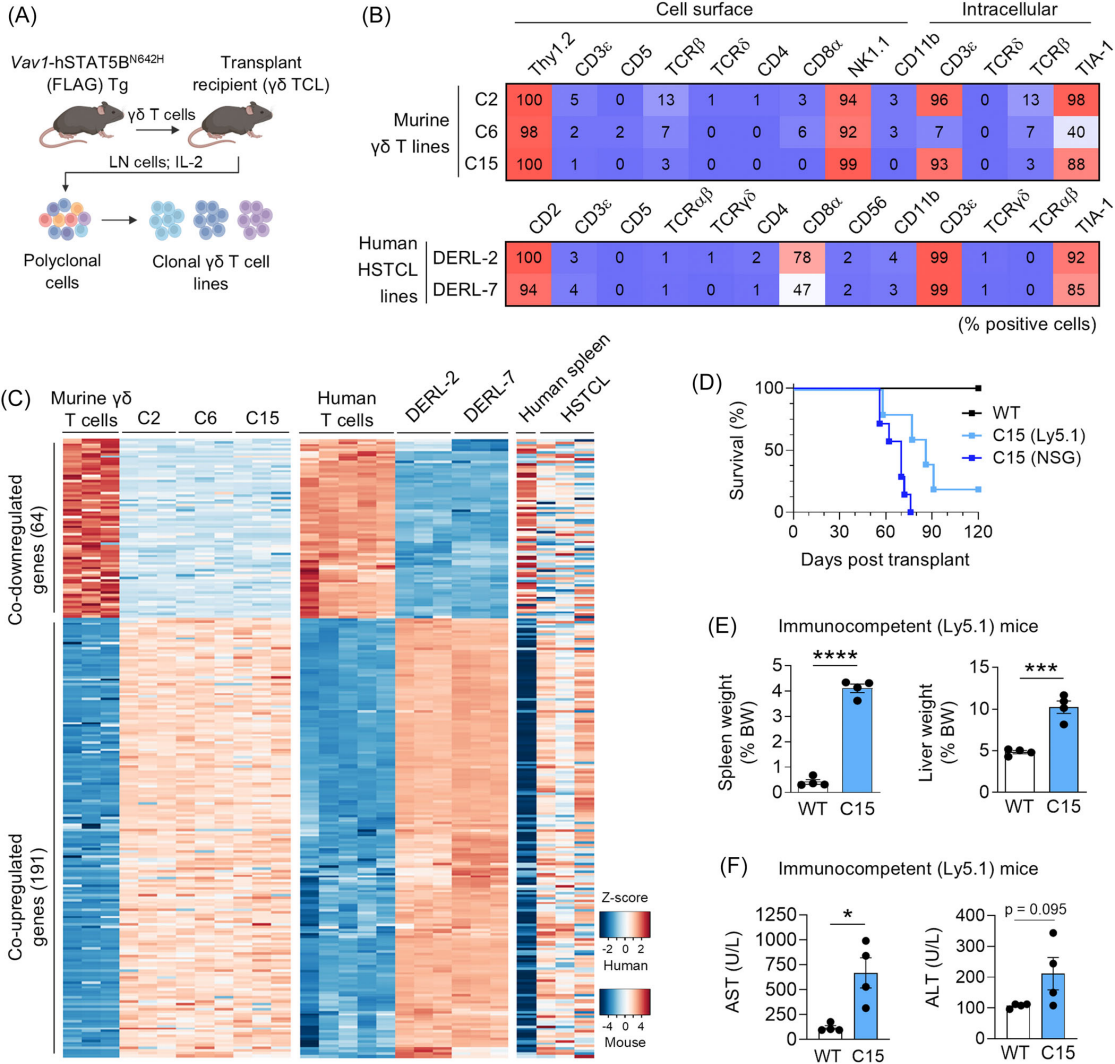
**Clonal, murine γδ T‐cell lymphoma cell lines expressing STAT5B^N642H^ display immunophenotypic and transcriptomic features of hepatosplenic T‐cell lymphoma (HSTCL), and can be allografted to generate an HSTCL‐like disease in mice. (A)** Schematic depicting the procedure taken to generate clonal murine γδ T‐cell lines from a human (h) STAT5B^N642H^ transgenic (Tg) mouse.^10^ LN, lymph node; TCL, T‐cell lymphoma. **(B)** Heatmap displaying percentage expression of various cell surface and intracellular protein markers from murine C2, C6, and C15 cell lines and from human DERL‐2 and DERL‐7 HSTCL cell lines, measured by flow cytometry. Data are displayed as average values from three independent experiments (*n* = 3). **(C)** Heatmap of normalized expression (*Z*‐scores) for commonly differentially expressed genes (DEGs) in murine C2, C6, and C15 cell lines (*n* = 3 each) compared to wild‐type (WT) C57BL/6 γδ T cells (*n* = 3), human DERL‐2 and DERL‐7 cell lines (*n* = 3 each) compared to normal human T cells (*n* = 5), and primary human HSTCL patient samples (*n* = 3) compared to healthy spleen (*n* = 1), determined by RNA‐seq (primary sample data were previously reported^11^). Genes were filtered for human–mouse homologs. **(D)** Kaplan–Meier survival analysis of C57BL/6 Ly5.1 (*n* = 5) or NSG (*n* = 7) mice transplanted with C15 cells via tail vein injection, or control (WT) mice (*n* = 4 Ly5.1; *n* = 3 NSG). **(E)** Spleen and liver weights (as % of body weight, BW) of WT and diseased C15‐recipient Ly5.1 mice. Data are graphed as mean (±SEM). ***P < 0.001, ****P < 0.0001; unpaired two‐tailed Student's *t*‐test. **(F)** Levels of AST and ALT in the plasma of WT and diseased C15‐recipient Ly5.1 mice. Data are graphed as mean (±SEM). *P < 0.05; unpaired two‐tailed Student's *t*‐test.

Despite that the N642H mutation stabilizes STAT5B tyrosine‐699 phosphorylation,^9^ STAT5 activation in the murine and human cell lines remained IL‐2 dependent, as shown by a loss of pY‐STAT5 upon IL‐2 withdrawal and restoration after IL‐2 re‐stimulation (Figure S1D,E). IL‐2 withdrawal was paralleled by a loss of proliferative capacity in the murine lines (Figure S1F), consistent with observations in the human HSTCL lines (Figure S1G),^10^ indicating a dependence on sustained IL‐2‐mediated oncogenic STAT5B activity. To confirm dependence on the driver mutation, we performed CRISPR‐Cas9‐mediated knockdown of the *STAT5B* ^*N642H*^ transgene in C15 cells (Figure S1H,I). Reduced STAT5B^N642H^ levels resulted in significant depletion of the cells over time compared with cells expressing a non‐targeting control sgRNA (Figure S1J).

The immunophenotype of the murine cells resembled the human HSTCL lines, including surface expression of Thy1.2/CD2 and intracellular CD3ε and TIA‐1 (Figure 1B). All lines lacked surface CD3ε, CD5, TCRδ, TCRβ, CD4, and CD11b, as well as intracellular TCRδ and TCRβ. While a lack of surface CD3ε and γδTCR is uncharacteristic of primary HSTCL,^1^ it is consistent with the reported immunophenotype of the DERL cells,^10^ suggesting in vitro downregulation/internalization of these markers. HSTCL tumor cells are typically CD8^−^, although CD8 expression has been reported.^1^ The DERL cells displayed variable CD8 expression, and the murine cell lines were consistently CD8^−^. Surprisingly, the DERL cell lines were CD56^−^ despite originating from a patient with CD56^+^ disease.^10^ The murine cell lines consistently expressed NK‐cell marker NK1.1 (Figure 1B).

To assess similarities in transcriptional profiles, we performed RNA‐seq of the murine and human cell lines and integrated publicly available RNA‐seq data from three primary HSTCL patient samples and one healthy control spleen sample.^13^ Differential gene expression analysis of the tumor cells compared to respective healthy controls, filtered for human–mouse homologs, revealed a considerable number of similarly dysregulated genes; a total of 255 overlapping differentially expressed genes were observed, including 191 genes co‐upregulated and 64 genes co‐downregulated across the mouse and human cell lines and the primary HSTCL patient tumors (Figure 1C). Gene set enrichment analysis revealed common significantly dysregulated pathways, with upregulated signatures including cell cycle, G2/M checkpoint, and E2F targets (Figure S2A,B). The cell cycle was previously identified as the top‐enriched pathway of upregulated genes in HSTCL tumor cells compared with normal γδ T cells.^14^ In contrast, commonly downregulated pathways included p53 targets and lymphocyte activation (Figure S2A).

Given the reported downregulation of cytotoxic factors in HSTCL tumor cells,^14^ we assessed the cytolytic potential of the murine γδ T‐cell lines. All three cell lines displayed minimal cytotoxicity against target YAC‐1 tumor cells (Figure S2C).

We next generated an HSTCL‐like in vivo allograft model by intravenous transplantation of C15 cells (Ly5.2^+^) into immunodeficient (NSG) or immunocompetent (Ly5.1) mice. NSG recipients developed γδTCL with 100% penetrance and a median survival of 70 days, and Ly5.1 mice showed 80% penetrance and a median survival of 86 days (Figure 1D). Both models displayed prominent hepatosplenomegaly (Figures 1E and S3A). Focusing on the immunocompetent model, we confirmed an expansion of tumor cells expressing Ly5.2, TCRδ, CD3ε, FLAG, and Ki67 in the liver (Figure S3B–D). Despite being absent in vitro, CD3ε and TCRδ were re‐expressed on the surface of the tumor cells in vivo (Figure S3E), consistent with primary HSTCL.^1^ Ly5.2^+^ tumor cells were detected in PB from week 7 post‐transplant (Figure S3F). Plasma ALT and AST liver transaminase levels in C15‐recipient mice were also elevated (Figure 1F). Histology revealed disrupted splenic architecture and atrophy of the white pulp (Figure S3G). Tumor cell infiltration was most prominent in the spleen, liver, BM, lymph nodes, and lung, and minimal in the kidney, brain, and heart (Figure S3H).

Whilst STAT5B^N642H^ is more persistently activated than WT STAT5B,^9^ it is not “constitutively” active, remaining dependent on upstream cytokine and JAK signaling.^4^, ^15^ Thus, we reasoned that JAKi could serve as promising targeted therapies in *STAT5B*‐mutated HSTCL, representing one‐third of HSTCL cases. RNA‐seq data revealed expression of all four JAK kinases in the murine and human HSTCL lines, with the highest expression of *Jak1* and *Jak3* in the murine cells and *JAK1* in the human cells. *Jak2*/*JAK2* was lowly expressed in all cell lines (Figure S4A). We tested eight JAKi with varying selectivity profiles (Figure 2A). All JAKi reduced HSTCL cell viability after 48 h, with selectivity over HH and Karpas 384 TCL lines that lack *JAK‐STAT* mutations^11^ and display no significant STAT5 activity (Figures 2A and S4B,C). As a control, the PARP inhibitor olaparib had minimal effects on HSTCL cell viability (Figures 2A and S4B). Notably, the efficacy of different JAKi varied by up to 100‐fold, with JAK1/2/3 inhibitor upadacitinib most potently inducing cell death and JAK1‐selective inhibitor abrocitinib displaying the weakest effects. Upadacitinib reduced the viability of all HSTCL lines with IC_50_ values of 27–102 nM (Figures 2A and S4B), accompanied by dose‐dependent suppression of STAT5 activation (Figure S4D), confirming the capacity of JAKi to block activation of the STAT5B oncogene.

**Figure 2:**
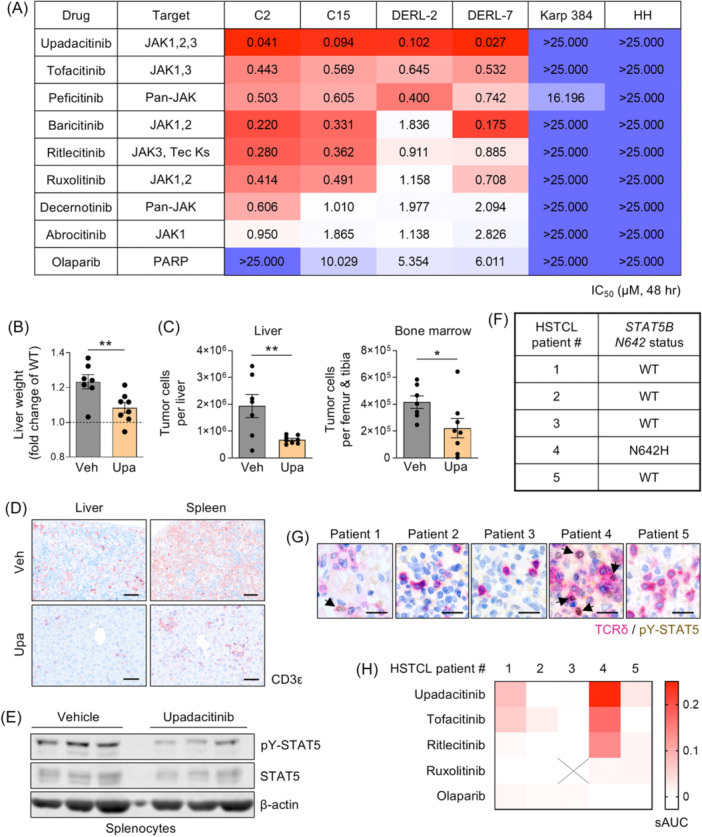
**JAK1/2/3 inhibitor, upadacitinib, displays anti‐tumor efficacy in hepatosplenic T‐cell lymphoma (HSTCL) cell lines, in the C15 allograft in vivo model, and in primary HSTCL patient cells harboring the *STAT5B* ^*N642H*^ mutation. (A)** Heatmap of IC_50_ values calculated from drug–response analyses using CellTiter‐Blue viability assays upon 48 h treatment of the indicated drugs in IL‐2‐supplemented media. Average IC_50_ values from three independent experiments are shown (*n* = 3). Tec Ks, Tec kinases. **(B)** Liver weights of C15‐recipient NSG mice treated with vehicle (veh; *n* = 7) or upadacitinib (upa; *n* = 8), graphed as fold change of the average liver weight of wild‐type (WT) NSG mice. All mice were analyzed 56 days post‐transplant. Data are graphed as mean (±SEM). **P < 0.01; unpaired two‐tailed Student's *t*‐test. **(C)** Absolute tumor cell numbers (intracellular FLAG^+^ cells) in the liver and bone marrow of vehicle or upadacitinib‐treated C15‐recipient NSG mice, analyzed by flow cytometry. Data are graphed as mean (±SEM). *P < 0.05, **P < 0.01; unpaired two‐tailed Student's *t*‐test. **(D)** Representative images from immunohistochemistry (IHC) analysis of liver and spleen sections from vehicle or upadacitinib‐treated C15‐recipient NSG mice, stained for CD3ε and imaged by light microscopy at 20× magnification (scale bar = 50 μm). **(E)** Western blot showing STAT5 activity in splenocytes from vehicle or upadacitinib‐treated C15‐recipient NSG mice (*n* = 3; three individual mice are shown per treatment group, representative of all mice from each group). Immunoblotting for pY‐STAT5 and total STAT5 was performed, with β‐actin serving as a loading control. **(F)** Table indicating the *STAT5B* mutation status at position N642 of HSTCL patients (*n* = 5), determined by digital polymerase chain reaction (PCR). **(G)** Representative images from IHC analysis of TCRδ (magenta) and pY‐STAT5 (brown) double staining in the bone marrow (Patient 1) or spleen (Patients 2–5) of HSTCL patients, imaged by light microscopy (scale bar = 20 μm). Black arrows indicate tumor cells with TCRδ and pY‐STAT5 double staining. **(H)** Heatmap of selective area under the curve (sAUC) values calculated from drug–response viability analyses using flow cytometry upon 24 h treatment of primary HSTCL patient tumor and non‐malignant cells with the indicated drugs (concentration range: 0.001–10 µM). A 24‐h incubation time was used for primary samples due to a considerable loss of cell viability of vehicle‐treated patient cells beyond 24 h of ex vivo culture. Average AUC values of healthy and tumor cell populations from technical duplicate samples were used. Ruxolitinib could not be tested on cells from Patient 3 (black cross) due to insufficient material.

We selected the top‐performing JAKi upadacitinib for in vivo testing using our HSTCL‐like preclinical model. NSG mice transplanted with C15 cells were treated orally with upadacitinib (10 mg/kg) or vehicle, beginning 10 days post‐transplant. At 8 weeks, a vehicle‐treated mouse displayed terminal disease symptoms, at which point all mice were sacrificed. Endpoint analysis showed reduced liver and spleen weights and reduced tumor burden in the liver, BM, spleen, and PB in upadacitinib‐treated mice (Figures 2B–D and S5A,B), accompanied by reduced STAT5 activation in splenocytes (Figure 2E). The drug was well tolerated, and no toxicity‐related impact on hemoglobin or platelet levels was observed (Figure S5C).

Finally, we assessed JAKi sensitivity in primary HSTCL patient samples. Both viable and FFPE samples from five patients were analyzed (Table S1). We screened the patient material for *STAT5B* ^*N642H*^ by digital polymerase chain reaction (PCR), first confirming the sensitivity and selectivity of the assay to the *STAT5B* ^*N642H*^ mutation using DERL‐2, DERL‐7, and Karpas 384 cell lines (Figure S6A–C). One out of the five HSTCL patients (20%) harbored the *STAT5B* ^*N642H*^ mutation (Figures 2F and S6D). We also assessed STAT5 activation in the patient tumor cells by immunohistochemistry (IHC) using pY‐STAT5/TCRδ double staining. Patient 4 (*STAT5B* ^*N642H*^) displayed clear nuclear STAT5 activity, Patient 1 showed weak STAT5 activity in some tumor cells, and no STAT5 activity was detected in the samples from Patients 2, 3, and 5 (Figures 2G and S7). We incubated viable HSTCL patient samples with different JAKi (upadacitinib, tofacitinib, ritlecitinib, or ruxolitinib) ex vivo for 24 h and measured cell viability using flow cytometry. The samples included both tumor and non‐malignant microenvironmental cells, distinguishable using defined surface markers (Table S1). Selective area under the curve (sAUC) values (tumor cell AUC minus healthy cell AUC) were calculated to reveal selective anti‐tumor effects. Strikingly, upadacitinib, tofacitinib, and ritlecitinib induced selective killing of *STAT5B* ^*N642H*^‐positive tumor cells (Patient 4), with the strongest effect observed for upadacitinib (Figure 2H), which induced cell death in >40% of tumor cells at 100 nM by 24 h (Figure S8A). Patient 1 showed modest sensitivity towards upadacitinib and tofacitinib, albeit less than Patient 4. Interestingly, ruxolitinib had minimal impact on tumor cell viability across all patient samples. There was no impact of JAKi on tumor cells from the other *STAT5B* WT patients or on healthy cells from all patients (Figures 2H and S8A–D). As a control, olaparib did not impact the viability of any patient cell population (Figures 2H and S8E).

In summary, we have developed a STAT5B‐driven mouse model using cell line‐based allografts, which recapitulates key features of HSTCL. Using this in vivo model, together with patient‐derived HSTCL cell lines and primary samples, we demonstrate selective anti‐tumor efficacy of upadacitinib against HSTCL cells with an activating *STAT5B* mutation, warranting biomarker‐driven clinical evaluation of upadacitinib in HSTCL patients. We also observed modest sensitivity to JAKi of tumor cells from Patient 1, who was negative for this mutation but displayed residual nuclear STAT5 activity. Therefore, screening STAT5 activity in HSTCL cells may represent a beneficial marker in addition to *STAT5B* mutation status to predict patient response to JAKi, potentially extending benefit to additional *STAT5B* WT patients. The number of HSTCL patient samples used in this study is low, reflecting the rarity of the disease, necessitating further preclinical and clinical validation.

With the high frequency of JAK‐STAT pathway hyperactivation in HSTCL and across most T‐cell lymphoma entities, interest in targeted therapies to block this pathway is increasing.^16^ However, the use of JAKi, particularly upadacitinib, in treating HSTCL patients remains largely unexplored. Moskowitz et al. described a Phase II biomarker‐driven clinical trial assessing the efficacy of JAK1/2 inhibitor ruxolitinib in patients with various peripheral T‐cell leukemias/lymphomas (PTCLs) harboring *JAK‐STAT* mutations.^17^ Overall, a clinical benefit was achieved in 53% of treated PTCL patients. Two HSTCL patients were included in this study, both with *STAT5B* ^*N642H*^ mutations, and both patients experienced disease progression within 6 months.^17^ In our study, ruxolitinib displayed relatively low anti‐tumor activity across all HSTCL cell lines and primary samples, in contrast to upadacitinib, which exhibited nanomolar‐range efficacy against *STAT5B*‐mutated HSTCL cells. Upadacitinib is widely reported as a JAK1‐selective inhibitor. However, studies comparing JAK family specificity have shown inhibition of JAK2‐ and JAK3‐dependent signaling by upadacitinib at clinically relevant doses.^18^, ^19^ Accordingly, our results suggest that JAKi with the strongest anti‐tumor activity against *STAT5B*‐mutated HSTCL inhibit both JAK1 and JAK3. This is perhaps expected given the important role of JAK1/3 in mediating lymphocyte proliferation and homeostasis downstream of IL‐2 and other cytokines acting via the common γ‐chain receptor.^20^ Further studies are required to dissect the relative contributions of JAK1 and JAK3 in HSTCL pathogenesis.

Overall, JAKi represent promising targeted therapy options for HSTCL with hyperactive STAT5, yet substantial variability in the efficacy of different JAKi is observed. This should be considered for future biomarker‐driven trials involving HSTCL patients, and potentially other PTCLs with frequent STAT5 activation, with upadacitinib emerging as a lead candidate.

## AUTHOR CONTRIBUTIONS

**Myint Myat Khine Aung**: Conceptualization; investigation; methodology; validation; formal analysis; funding acquisition; visualization; project administration; writing—original draft; writing—review and editing; software. **Susann Schönefeldt**: Investigation; validation; formal analysis; writing—original draft; writing—review and editing; visualization; methodology. **Sophie Pfalz‐Kraupp**: Investigation; methodology; validation; writing—review and editing; visualization; formal analysis. **Tamara Wais**: Investigation; methodology; visualization; writing—review and editing; formal analysis. **Tobias Suske**: Investigation; writing—review and editing; methodology. **Safia Zahma**: Investigation; methodology; writing—review and editing; formal analysis. **Stefan Franz**: Investigation; methodology; writing—review and editing; formal analysis. **Andrea Müllebner**: Investigation; methodology; validation; visualization; writing—review and editing; formal analysis. **Gabriela Feurstein**: Investigation; writing—review and editing; methodology; formal analysis. **Christina Wagner**: Investigation; methodology; writing—review and editing; formal analysis. **Thomas Eder**: Investigation; methodology; visualization; writing—review and editing; software; formal analysis; data curation. **Tea Pemovska**: Investigation; writing—review and editing; visualization; methodology; formal analysis. **Christiane Agreiter**: Investigation; methodology; writing—review and editing; formal analysis. **Alexander Pichler**: Investigation; methodology; writing—review and editing; formal analysis. **Philipp B. Staber**: Resources; supervision; writing—review and editing; funding acquisition. **Martin Hofer**: Investigation; methodology; writing—review and editing; formal analysis. **Ralf Steinborn**: Methodology; investigation; writing—review and editing; formal analysis; supervision. **Svenja‐Verena Strohmer**: Investigation; methodology; writing—review and editing. **Ingrid Simonitsch‐Klupp**: Investigation; writing—review and editing; visualization; methodology; formal analysis; resources; supervision. **Marcus Bauer**: Investigation; methodology; visualization; writing—review and editing; formal analysis. **Andreas Wilfer**: Investigation; methodology; writing—review and editing; formal analysis. **Thomas Weber**: Investigation; methodology; writing—review and editing; supervision. **Swapnil Potdar**: Investigation; methodology; writing—review and editing; software; formal analysis. **Tero Aittokallio**: Funding acquisition; writing—review and editing; supervision. **Dennis Jungherz**: Investigation; methodology; writing—review and editing; formal analysis. **Tony A. Müller**: Investigation; methodology; writing—review and editing. **Julia List**: Investigation; methodology; writing—review and editing; formal analysis. **Dagmar Gotthardt**: Investigation; methodology; writing—review and editing; supervision; funding acquisition. **Florian Grebien**: Writing—review and editing; supervision. **Vasileios Bekiaris**: Writing—review and editing; methodology; supervision; formal analysis. **Marco Herling**: Funding acquisition; writing—review and editing; supervision; resources. **Richard Moriggl**: Supervision; writing—review and editing; funding acquisition. **Heidi A. Neubauer**: Conceptualization; writing—original draft; funding acquisition; writing—review and editing; supervision; project administration; formal analysis; visualization; methodology; data curation; resources.

## CONFLICT OF INTEREST STATEMENT

A.P. reports being an employee, co‐founder, and shareholder of exalt®FlexCo, which uses techniques described in this manuscript for translational research. Data interpretation was performed while being a PhD student at the Medical University of Vienna. A.P. reports, unrelated to the submitted work, a grant from the Austrian Society of Hematology and Oncology and personal fees from Takeda. T.P. reports being a founder and shareholder of exalt®FlexCo and has pending patents EP24158079.4 and EP24169882.8, licensed to the Medical University of Vienna. All other authors declare no competing financial or any other relevant conflicting interests.

## FUNDING

M.M.K.A., S.P.‐K., T.W., C.W., R.M., and H.A.N. were supported by the Austrian Science Fund (FWF) grant SFB‐F06109, H.A.N. was additionally supported by the FWF grant P 35378‐B, and J.L. and D.G. by the FWF grant ZK‐81B (DOI: 10.55776/ZK81). S.S., T.A., M.He., and H.A.N. were supported by their local funding agencies under the frame of ERA PerMed (JAK‐STAT‐TARGET). M.He., P.B.S., and R.M. were supported under the frame of ERA‐NET (ERANET‐PLL). P.B.S. was supported under the frame of ERA‐NET on Translational Cancer Research (TRANSCAN‐2; EuroTCLym). H.A.N., S.P.‐K., M.He., and T.A. were supported under the frame of EP PerMed (ImmuneT‐ME). M.M.K.A. was additionally supported by an Austrian Academy of Sciences (ÖAW) DOC Fellowship. T.P. was supported by a grant from Krebsforschungslauf and the Initiative Krebsforschung (Comprehensive Cancer Center Vienna‐Medical University of Vienna). V.B. was supported by Kræftens Bekæmpelse (R269‐A15747), the Novo Nordisk Foundation (NNF20OC0065160), and the Lundbeck Foundation (R366‐458 2021‐104). For open access purposes, the authors have applied a CC BY public copyright licence to any author‐accepted manuscript version arising from this submission. Open Access funding provided by Veterinarmedizinische Universitat Wien/KEMÖ.

## Supporting information

Supporting Information.

## Data Availability

The RNA sequencing data reported in this article have been deposited in the GEO database under the accession number GSE271616. All other relevant data that support the conclusions of the study are available on request from the corresponding author, Heidi Neubauer (heidi.neubauer@vetmeduni.ac.at).
