# Supplementary material for: Preclinical models of hepatosplenic γδ T‐cell lymphoma with an activating STAT5B mutation display sensitivity to JAK inhibitor upadacitinib
Source: Hemasphere. 2026 Apr 16;10(4):e70345. doi: 10.1002/hem3.70345 (PMC13084257; doi:10.1002/hem3.70345)
Supplement: Supplementary file 1 — Supporting Information. [file HEM3-10-e70345-s001.pdf]

Supplementary Information

Preclinical models of hepatosplenic γδ T-cell lymphoma with an activating STAT5B mutation display sensitivity to JAK inhibitor upadacitinib

Supplementary Methods

Study approvals

All mouse breeding and experiments were authorized by the Austrian Federal Ministry of Education, Science and Research (BMBWF-68.205/0084-V/3b/2019, 2023-0.651.094, 2024-0.191.292, BMWFW-68.205/0093-WF/V/3b/2015 and 2022-0.404.452). Collection and use of healthy-donor derived T cells isolated from buffy coats for RNA-seq were approved for research purposes by the ethics committees of the University Hospital of Cologne (#19-1089). Viable, cryopreserved samples from the Viennese Viable Biobank for Haematological Diseases (Vivibank, EK 1284/18) and associated FFPE tumor samples from HSTCL patients were obtained with approval from the ethics committees of the Medical University of Vienna (EK 1448/2024). The research was conducted in accordance with the Declaration of Helsinki.

hSTAT5B^{N642H} transgenic mouse model

The *Vav1*-hSTAT5B^{N642H} transgenic mouse model, from which the clonal γδ T-cell lines were derived, was previously generated as described elsewhere.[1] Briefly, the *Vav1*-hematopoietic vector containing human STAT5B^{N642H} with a C-terminal FLAG-tag was digested with the *HindIII* restriction enzyme and gel purified for pronuclear injection into C57BL/6NCrl mice. The transgenic mice were identified by genotyping PCR and confirmed to express low-level (2x endogenous STAT5 levels), strongly activated STAT5B protein by Western blot of lymph node and spleen. Given the rapid development of CD8$^+$ T-cell disease in hSTAT5B^{N642H} transgenic mice, the colony must be propagated via *in vitro* fertilization with archived sperm cells.

Generation of clonal murine cell lines

Wildtype C57BL/6N mice transplanted with γδ T cells isolated from lymph nodes of *Vav1*-hSTAT5B^{N642H}(FLAG) transgenic mice developed a γδ T-cell lymphoma/leukemia

($CD3^+\gamma\delta TCR^+$) phenotype, as previously reported.[2] Cryopreserved lymph node cells from one terminally diseased mouse were thawed and $2x10^6$ cells were incubated at 37°C in 5 ml media containing RPMI 1640 (Gibco) with 20% heat-inactivated fetal bovine serum (hi-FBS; Biowest), 2mM L-glutamine (Gibco), 10 U/ml penicillin/streptomycin (Biowest), 55 µM β-mercaptoethanol (Gibco) and 40 ng/ml recombinant human interleukin-2 (IL-2; ImmunoTools GmbH). Every seven days, 2.5 ml of the media was removed and replaced with 2.5 ml fresh media containing 110 µM β-mercaptoethanol and 80 ng/ml IL-2. After two weeks, large, proliferative cells with mostly adherent properties were observed and expanded as detailed below. Clonal lines were further generated by FACS sorting single-cells into a 96-well plate using a FACSAria III cell sorter (BD Biosciences) and expanding in standard media with 10 ng/ml IL-2 and Normocin™ (Invitrogen). The clonal lines were cultured in the presence of Normocin™ until they had been expanded into 25 cm² cell culture flasks, to minimise the risk of bacterial contamination.

Cell culture

Murine γδ T-cell lines were cultured in media containing RPMI 1640 supplemented with 20% hi-FBS (Biowest), 2 mM L-glutamine (Gibco), 10 U/ml penicillin/streptomycin (Biowest), 1X MEM Non-Essential Amino Acids Solution (Gibco), 20 mM HEPES buffer (Gibco), 1 mM sodium pyruvate (Gibco), 55 µM β-mercaptoethanol (Gibco) and 10 ng/ml IL-2 (ImmunoTools GmbH). The cell lines were sub-cultured every 2-3 days in fresh media using a standard trypsinization protocol. DERL-2 and DERL-7 human HSTCL cell lines and the HH cutaneous T-cell lymphoma cell line were obtained from the German Collection of Microorganisms and Cell Cultures GmbH (DSMZ). The Karpas 384 primary cutaneous γδ T-cell lymphoma cell line was obtained from the European Collection of Cell Cultures (ECACC). All human cell lines were cultured in media containing RPMI 1640 supplemented with 10% hi-FBS, 10 U/ml penicillin/streptomycin and 2 mM L-glutamine. DERL-2 and DERL-7 culture media was additionally supplemented with 10 ng/ml IL-2 (ImmunoTools GmbH). The murine YAC-1 lymphoma cell line was purchased from the American Type Culture Collection (ATCC) and was cultured in RPMI 1640 media including 10% FBS, 10 U/ml penicillin/streptomycin and 55 µM β-mercaptoethanol. Primary mouse NK cells were expanded in RPMI 1640 media

containing 10% FBS, 10 U/ml penicillin/streptomycin, 55 µM β-mercaptoethanol and 3000 U/ml recombinant human IL2 (Proleukin, Sterimax).

All cell lines were incubated at 37°C with 5% CO_2 in a humidified environment and were regularly tested and confirmed negative for mycoplasma using PhoenixDx Mycoplasma Mix (Procomcure Biotech). The authenticity of the DERL-2, DERL-7, HH and Karpas 384 cell lines was confirmed by short tandem repeat (STR) profiling (Microsynth GmbH). Upon thawing cryopreserved cell lines, new stocks were prepared and frozen within 3-5 passages. Cell lines were kept in culture for no more than two months after resuscitation.

For cytokine starvation experiments, murine γδ T-cell lines were cultured for 48 hr in standard media with 10 ng/ml IL-2 (pre-starvation). Cells were then washed three times with Dulbecco's Phosphate-Buffered Saline (DPBS; Sigma-Aldrich) and cultured for an additional 8 hr in media without IL-2 (starvation). Cells were re-stimulated for 1 hr with 10 ng/ml IL-2 (re-stimulation). From all conditions, a cell pellet was collected for Western blot analysis.

Western blotting

For immunoblotting of cytokine-dependent cell lines, cells were stimulated with 10 ng/ml fresh IL-2 for 2 hr prior to harvesting cell pellets. For Western blot analysis of upadacitinib-treated cell lines, cells were exposed to increasing concentrations of upadacitinib or DMSO vehicle for 4 hr. During this period, cells were starved of IL-2 for the first 3.5 hr, followed by 10 ng/ml IL-2 restimulation for the final 30 min. For Western blotting of primary mouse splenocytes, erythrocyte lysis was performed prior to collection of splenocyte pellets. 30 µg total protein was subjected to immunoblotting using standard techniques. Nitrocellulose membranes (0.45 µm Cytiva Amersham Protran, Fisher Scientific) were blocked in Odyssey Blocking Buffer (Intercept TBS Blocking Buffer, LI-COR Biosciences) and incubated with the respective antibody diluted in the same buffer. Details of the antibodies used for Western blotting are available in **Table S2**. Images were obtained using IRDye fluorescent secondary antibodies and an Odyssey CLx imaging system (LI-COR Biosciences). The signal intensities of the bands were quantified via densitometry using Image Studio Lite v5.2 software (LI-COR Biosciences).

STAT5B^{N642H} gene editing and cell competition assay

For CRISPR-Cas9-mediated editing of the human *STAT5B^{N642H}* transgene, C15 cells were lentivirally transduced with a Cas9- and GFP-expressing LentiCRISPRv2GFP vector (#82416; Addgene) additionally containing human *STAT5B* single guide RNAs (sgRNAs) or non-targeting control sgRNAs (sequences listed in **Table S3**). To confirm knockdown of the STAT5B^{N642H} oncogene, cell pellets of transduced (GFP^{+} sorted) cells were harvested at day 4 or 11 post-transduction for analysis of mRNA and protein levels. To assess *STAT5B* mRNA levels, RNA was isolated using a Monarch® Total RNA Miniprep Kit (New England Biolabs) and was used to generate cDNA using a RevertAid First Strand cDNA Synthesis Kit (Thermo Fisher Scientific) according to the manufacturer's protocol. Gene expression was analyzed by qRT-PCR using a GoTaq® qPCR Master Mix (Promega) according to the manufacturer's protocol, and a CFX Connect Real-Time PCR Detection System (Bio-Rad). Relative mRNA levels were analyzed using the $2^{-\Delta\Delta Ct}$ method and normalized to *GAPDH* gene expression. Primers are listed in **Table S4**. To assess STAT5B^{N642H} protein levels, 30-40 µg total protein was extracted and subjected to immunoblotting with antibodies against the FLAG-tag (**Table S2**), as described above.

For the cell competition assay, the population of C15 cells successfully transduced with sgRNAs (percentage of GFP^{+} cells) was monitored over time using an iQue3 flow cytometer (BioScience, Sartorius Group) and normalised to day 3 post-transduction and to the non-targeting control (NTC)-1.

Sanger Sequencing

Cells were harvested and genomic DNA was isolated using standard protocols. DNA concentration and purity were determined using a NanoDrop 2000 spectrophotometer (Thermo Fisher Scientific). A sequence encoding the STAT5B C-terminal region was amplified by PCR. The 646 bp product was separated by agarose gel electrophoresis and isolated using a MinElute PCR Purification Kit (Qiagen). Sanger sequencing was carried out by Microsynth Austria GmbH. All primer sequences are listed in **Table S4**.

Proliferation assay

C2, C6 and C15 cells were seeded at $1x10^4$ cells/well, and DERL-2 and DERL-7 cells were seeded at $2x10^4$ cells/well, into 24-well plates. Each cell line was seeded in the presence or absence of 10 ng/ml IL-2 and in technical triplicates for each time point. At the respective time points, cells were harvested into FACS tubes, washed once with PBS, and cell numbers were quantified with the addition of Precision Counting Beads (Biolegend) using a BD FACSCanto II flow cytometer, with FACSDiva (BD Biosciences) and FlowJo (version 10.5.3) software.

RNA sequencing

For murine samples: C2, C6 and C15 cell lines were cultured in biological triplicates and harvested by trypsinization. Primary murine γδ T cells from three individual female WT C57BL/6N mice were isolated using lymph node single cell suspensions. Murine cells were stained with FACS antibodies to discriminate viability dye$^-$ Ter119$^-$ TCRβ$^-$ CD45.2$^+$ TCRδ$^+$ cells (see **Table S5**). 500 cells/well were collected from all murine samples by FACS sorting, using a FACSAria III cell sorter, into a hard-shell, low-profile, thin-wall 96-well skirted PCR plate (Bio-Rad) containing 4 µl lysis buffer per well (2 U/µl RNase inhibitor (Clontech) in 0.2% (v/v) Triton X-100). Plates were kept at -80°C until further processing at the Biomedical Sequencing Facility (BSF; CeMM, Vienna, Austria). For preparing NGS libraries, we followed the Smart-seq2 protocol.[3] The subsequent library preparation from the amplified cDNA was performed using a Nextera XT DNA library prep kit (Illumina, San Diego, CA, USA). Library concentrations were quantified with a Qubit 2.0 Fluorometric Quantitation system (Life Technologies, Carlsbad, CA, USA) and the size distribution was assessed using a 2100 Bioanalyzer instrument (Agilent, Santa Clara, CA, USA). For sequencing, samples were diluted and pooled into NGS libraries in equimolar amounts and sequenced on a HiSeq 4000 instrument (Illumina) in 50 bp single end mode.

For human samples: EDTA peripheral blood mononuclear cells (PBMCs) of healthy human donors were isolated by density gradient centrifugation (#25-072-CV, Corning). CD3$^+$ T-cell enrichment from PBMCs was obtained using magnetic cell separation according to the

manufacturer's instructions (negative selection, #480021, Biolegend). DERL-2 and DERL-7 cell lines were cultured in biological triplicates, harvested, and RNA extraction was performed with an RNeasy Plus Micro kit (Qiagen) according to the manufacturer's protocol. RNA concentration and purity were measured with a NanoDrop 2000 spectrophotometer. RNA was subjected to polyA-based library preparation and sequenced on a NovaSeq 6000 platform (Illumina) according to the manufacturer's instructions.

PRINSEQ-lite[4] (version 0.20.4) was used for data quality filtering, trimming, and length filtering. High-quality reads were aligned using STAR[5] (version 2.7.9a) to the mouse (mm10) reference genome for Smart-seq2 datasets and to the human (GRCh38) reference genome for RNA-seq datasets. Aligned reads were subsequently processed with Samtools[6,7] (version 1.13). FeatureCounts[8] from the subread package (version 2.0.3) was used for counting reads per gene. Normalization and differential expression analysis were conducted using DESeq2.[9] For visualization, ggplot2[10] was used in R to create heatmaps. To facilitate cross-species analysis, Bioconductor libraries were used, including Orthology.eg.db,[11] org.Mm.eg.db[12] and org.Hs.eg.db,[13] for translating mouse gene identifiers to their human orthologs.

For patient HSTCL samples, publicly available RNA-seq data from the Gene Expression Omnibus dataset GSE57944[14] were extracted, including case 4, case 5, case 7 and normal spleen samples. These data were processed following the same pipeline described above for human RNA-seq data. To generate the combined heatmap, the ComplexHeatmap[15] package was applied using Z-scores of normalized expression values from all three datasets. The depicted gene list was obtained by identifying significantly differentially expressed genes (P-adj ≤ 0.05 and log2 fold change > 2 or < -2) between the three patient samples and the healthy human controls that had one-to-one orthologs in mouse and were also differentially regulated in the other two comparisons. Gene set enrichment analysis (GSEA)[16] was performed using these overlapping DEGs, pre-ranked by log2 fold change values, and gene sets obtained from the Human Collection Molecular Signatures Database (MSigDB), using GSEA software v4.4.0 (Broad Institute) with 1000 permutations. The enriched pathways were then visualized as a bubble plot with the size of each bubble representing -log10 False Discovery Rate (FDR), and

the colour corresponding to either up- or down-regulated pathways, using the Google Colaboratory platform. Additionally, GSEA was performed with normalized gene expression count lists from the murine and human cell lines and respective controls, obtained from the DESeq2 analysis, and gene sets (equivalent mouse and human collections) obtained from the MSigDB, using GSEA software v4.3.2 (Broad Institute) with 1000 permutations and 'gene_set' as the permutation type.

Cell killing assay

Murine YAC-1 lymphoma cells, used as target cells, were labelled with CSFE (Invitrogen) and $1x10^5$ cells were seeded per well into flat bottom 96-well plates. The clonal murine γδ T-cell lines were then added at the indicated effector:target cell ratios. Primary mouse NK cells, used as a positive control, were isolated and cultured in media supplemented with 3000 U/ml human IL-2 (Novartis), as previously described.[17] The plates were centrifuged at 11 *g* for 2 min to bring the cells into close proximity. After 4 hr incubation at 37°C, the specific lysis was assessed by flow cytometry using SYTOX™ Blue Dead Cell Stain (Invitrogen) to quantify lysed target cells. Percentage of specific lysis was calculated as follows: [% SYTOX+ CFSE+ cells after co-incubation with effector cells] – [% SYTOX+ CFSE+ cells without addition of effector cells (spontaneous lysis control)].

Cell line drug treatments

Drugs were purchased from MedChemExpress and reconstituted in DMSO. Cells were seeded in triplicates into flat-bottom 96-well plates (Greiner AG) at $2x10^4$ cells/well. The following day, cells were treated with serial two-fold or five-fold dilutions of the drugs, or DMSO as a negative control, in 10 ng/ml IL-2 supplemented media. Bortezomib (100 µM) served as a positive control. Cells were incubated at 37°C for 48 hr. Cell viability was measured using a CellTiter-Blue Cell Viability Assay (Promega) on a GloMax Discover Microplate Reader (Promega). IC_{50} values were determined using Breeze software.[18]

Drug sensitivity profiling in primary patient cells

Spleen, BM or PB samples from HSTCL patients (**Table S1**) were cryopreserved after Ficoll-Paque mononuclear cell separation. Cell viability profiling was performed as previously described.[19,20] After 24 hr drug treatment, cells were stained with DAPI and antibodies marking tumor or healthy cells, and multiplex cell analysis was performed using high-throughput flow cytometry.

Mouse strains and housing

C57BL/6 Ly5.1 (B6.SJL-Ptprc^{a}Pepc^{b}/BoyCrl) and *NOD-scid IL2Rgamma^{null}* (NSG) mice were obtained via in-house breeding at the University of Veterinary Medicine Vienna (Vienna, Austria). Additionally, NSG mice were purchased from Janvier Labs. Female mice between 8-12 weeks of age were used for the experiments in this study. Mice were maintained under specific pathogen-free (SPF) conditions in individually ventilated cages at the University of Veterinary Medicine Vienna. Mice were kept in a 12/12-hour light/dark cycle and received standard food and water *ad libitum*.

***In vivo* allograft models and JAK inhibitor treatment**

C15 cells were stimulated with 10 ng/ml IL-2 two hr prior to transplantation, and 1×10^{6} cells in 100 µl PBS were intravenously administered to C57BL/6 Ly5.1 or NSG mice via tail vein injection. Post C15 cell transplantation, recipient mice were monitored daily for physical signs of disease (hunching, slow movement, ruffled fur). The percentage of peripheral blood tumor cells, obtained from puncture of the *vena facialis*, was determined every two weeks via FACS analysis of CD45.2^{+} cells (in Ly5.1/CD45.1 recipients) or FLAG-tag^{+} cells (NSG recipients). Mice were considered to have reached end-stage disease when physical signs of disease reached ethical limits and/or the percentage of tumor cells in the blood reached 25%, at which point mice were humanely sacrificed. Directly prior, whole blood samples were collected via heart puncture into EDTA-treated tubes (MiniCollect® K3EDTA, Greiner Bio-One). Immediately following euthanasia, mice were subjected to whole-body perfusion with PBS to remove blood contamination from tissues.

For *in vivo* upadacitinib treatment, C15 cells were intravenously transplanted into 15 female NSG mice, aged 11-12 weeks, as described above. Ten days post-transplantation, recipient mice were randomly distributed into two groups and treatment commenced of once-daily oral gavage with either 10 mg/kg upadacitinib (MedChemExpress; n = 8) or vehicle (5% DMSO, 40% PEG-300, 5% Tween-80, 50% PBS; n = 7). Mice were treated five days per week for four weeks (cycle 1) followed by another two weeks of treatment (cycle 2), with a one-week break in treatment between cycles. Once any of the mice in the experiment reached end-stage disease (as determined by our termination criteria in accordance with ethical limits), all mice were sacrificed as described above and comparative end-point analyses were conducted.

Preparation of cells from mouse tissues

Single-cell suspensions were prepared from spleen and liver by crushing organs in ice-cold PBS through a 100 µm cell strainer (BD Biosciences). Bone marrow cells were harvested from the femur and tibia by cutting one end of the bone and centrifuging the contents at 2500 x *g* for 1 min into an Eppendorf tube containing plain RPMI media. Erythrocytes from spleen, bone marrow and peripheral blood were lysed using Ammonium-Chloride-Potassium (ACK) buffer (150 mM NH_4CO_3, 10 mM $KHCO_3$, 1 mM EDTA, pH 7.2). Liver cells were pelleted by centrifugation and then resuspended in 4 ml 40% Percoll® (Sigma-Aldrich), layered onto 4 ml 70% Percoll®, and centrifuged at 800 x *g* for 20 min at room temperature with deceleration set to 0. Leukocytes from the interphase were collected and washed once in RPMI 1640 media supplemented with 10% hi-FBS.

Blood biochemistry and hematocytometry

Whole blood from mice was collected in EDTA-treated tubes (MiniCollect® K3EDTA, Greiner Bio-One). Platelets and haemoglobin levels were measured from whole blood using an animal blood counter (Scil Vet ABC). To analyze alanine aminotransferase (ALT) and aspartate aminotransferase (AST) levels, whole blood was centrifuged at 5000 x *g* for 5 min at room temperature, plasma was collected and analyzed undiluted or at a 1:3 dilution in normal saline (0.9%) using an IDEXX Vet Test 8008 Veterinary Chemistry Analyzer.

Immunohistochemistry and H&E staining

Mouse organs were incubated for 24 hr in 4% phosphate-buffered formaldehyde solution (Roti-Histofix; Carl Roth), dehydrated, embedded and cut into 2.5 µm thick sections. Immunohistochemical (IHC) stainings of CD3, Ki67, FLAG and TCRδ, as well as hematoxylin and eosin (H&E) staining, were performed using standard protocols with the following specifications. Heat-mediated antigen retrieval was performed in citrate buffer at pH 6.0 (Dako) in an autoclave for 20 min (CD3, Ki67), or in a microwave for 7 min at 800 W and 15 min at 290 W (FLAG). CD3 staining (CD3ε rabbit mAb, #85061, Cell Signaling Technology; 1:300) and Ki67 staining (Ki67 rabbit mAb, #12202, Cell Signaling Technology; 1:1000) were performed with Mouse-To-Mouse Blocking Reagent (#MTM125, ScyTek Laboratories), secondary antibodies from an UltraTek HRP Anti-Polyvalent Staining Kit (#AFN600, ScyTek Laboratories) and AEC-Chromogen substrate (#ACD030, ScyTek Laboratories). FLAG staining (FLAG rabbit mAb, #14793, Cell Signaling Technology; 1:500) was performed with 5% goat-serum blocking, secondary antibodies from SignalStain Boost IHC Detection Reagent (HRP rabbit, #8114, Cell Signalling Technology) and ImmPACT DAB substrate kit (#SK-4105, Vector Laboratories). TCRδ staining (TCR δ H-41, #sc-100289, Santa Cruz Biotechnology; 1:100) was performed after heat-mediated antigen retrieval in TRIS-EDTA buffer at pH 9.0 for 20 min (#ZUC029-500, Zytomed Systems) and blocking with Mouse-To-Mouse Blocking Reagent (#MTM125, ScyTek Laboratories). Secondary antibody (#POLHRP-100, Zytomed Systems) and visualization (#DAB530, Zytomed Systems) were applied according to the manufacturer’s instructions. Hematoxylin (Mayer′s hemalum solution, #109249, Sigma-Aldrich) and Eosin G (C.I. 45380, #7089, Carl Roth) were applied according to the manufacturer's protocols. Sections were imaged using an Olympus BX53F2 LED light microscope with an Olympus SC50 camera or were scanned with an Evident SLIDEVIEW research slide scanner VS200 and analyzed with OlyVIA software (version 3.4.1, Olympus). Percentage of tumor cell organ infiltration was assessed using CD3 stained organ sections and scored across three mice per genotype by a trained pathologist in a blinded manner.

IHC analyses on human HSTCL tissue samples were performed on 2 μm sections of FFPE tissue using an automated BOND-III Immunostainer (Leica Biosystems). Heat induced epitope retrieval was performed with BOND Epitope Retrieval Solution (solution 2, Leica Biosystems #AR9640) for 20 min. Double IHC staining for pY-STAT5 and TCRδ was performed in a sequential manner, using primary antibodies against phospho-Stat5 (Tyr694, C11C5, rabbit mAb, Cell Signaling Technology #9359; 1:25) and TCRδ (H-41, mouse mAb, Santa Cruz Biotechnology #sc-100289; 1:300) incubated for 30 min each. pY-STAT5 was stained first and developed using a BOND Polymer Refine Detection Kit (brown color; Leica Biosystems #DS9800), followed by staining for TCRδ and developing with a BOND Polymer Refine Red Detection Kit (magenta color; Leica Biosystems #DS9390), according to the manufacturer's guidelines. Tissue sections were then counterstained with hematoxylin.

Flow cytometry

For flow cytometry analysis, cells were transferred to a V-bottom 96-well plate. Fc receptor was blocked with blocking antibody (diluted at 1:100) for 30 min in the dark, and cells were then incubated with antibodies against surface proteins (diluted at 1:200) for 1 hr at 4°C in the dark (**Table S5**). For intracellular staining, cells were resuspended in 100 μl of Fix/Perm solution (BD Biosciences) per well in a V-bottom 96-well plate for 20 min at 4°C. Subsequently, cells were washed twice in Perm/Wash Buffer (BD Biosciences), blocked with Fc receptor blocking antibody (diluted at 1:100) and then incubated with antibodies (diluted at 1:200; **Table S5**) in Perm/Wash Buffer following the same staining procedure as above. All analyses were performed on a BD FACSCanto II using FACSDiva (BD Biosciences) and FlowJo (version 10.5.3) software or on a Beckman Coulter Life Sciences CytoFLEX benchtop flow cytometer and CytExpert (version 2.4.0.28) software.

***STAT5B* SNP genotyping using digital PCR (dPCR)**

FFPE spleen or bone marrow tissue from five HSTCL patient tumors were cut into 10 μm sections, and 1-5 sections per tumor were collected into a 1.5 ml Eppendorf tube. Genomic DNA isolation was performed using an AllPrep® DNA/RNA FFPE isolation kit (QIAGEN),

according to the manufacturer's protocol. For the deparaffinization step, the protocol using heptane and methanol was used and was performed twice.

dPCR quantification was performed on the 3-colour naica® system (Stilla Technologies, Villejuif, France). The target of interest was a SNP causing the oncogenic somatic missense mutation N642H, located in exon 16 of human *STAT5B* (NM_012448.4: c.A1924C). Assay oligonucleotides were designed using Primer3 (version 2.5.0), integrated into NCBI's Primer-BLAST,[21] and the DNA sequence surrounding the SNP of interest (GenBank: NM_012448.4). To test for cross amplification, the RefSeq Representative Genome Database was restricted to *Homo sapiens*. Six locked nucleic-acid (LNA) monomers were introduced to increase structural stability of the SNP-specific probe. To enhance the disruptive effect of the single mismatch, the probe sequence was shortened to ten nucleotides and designed to melt at a temperature that was 5 to 7°C above the annealing/extension temperature of 60°C.[22] The melting temperature (*Tm*) was predicted using the *Tm* Prediction tool for LNA-enhanced oligonucleotides (Qiagen; https://geneglobe.qiagen.com/us/tools/tm-prediction). LNA monomers were inserted at the polymorphic site located in the probe centre and across the sequence body except for the 5′ terminal base. This allowed cleavage by the 5′ to 3′ nuclease activity of *Taq* DNA polymerase.[23] The SNP genotyping assay used the primers 5'-TGA TTG TTC TGT TTA TTG ATC TAG AGG and 5'-AAT GGA GAA GTC TCT GGT GGT AAA together with the Affinity Plus® hydrolysis probe 5'-FAM-CA+G+A+T+<u>G</u>+C+CAA/TAO™/A-Iowa Black™ RQ, where the substitution is underlined and the "+" sign precedes an LNA base. The number of haploid genomes was counted using an 87-bp-amplicon assay targeting the single-copy gene *RPP30*,[24] with fluorescence generation achieved by a Cy5-labeled double-quenched probe incorporating the internal TAO™ Quencher and the 3' Iowa Black RQ®. Oligonucleotides were synthesised at Integrated DNA Technologies (Leuven, Belgium). The 25 µl reaction volume consisted of 10× naica® Multiplex PCR Mix (2.5 µl buffer A and 1 µl buffer B; Stilla Technologies), 800 nM of each primer, 300 nM of each probe and 2 µl DNA. The reaction mixture was loaded on a Sapphire chip capable of forming up to 30,000 droplets per sample. The chip was placed into the Geode instrument for partitioning the reaction into

droplets, formation of droplet crystals and amplification. Amplification was performed according to the following parameters: initial denaturation step at 95°C for 3 min, 45 cycles at 95°C for 10 sec and 60°C for 40 sec. Fluorescence images were obtained using the naica® Prism3 fluorescence reader. Spill-over compensation was performed manually and was applied before data analysis with the Crystal Miner™ software version 4.0.10.3 (Stilla Technologies).

Accuracy of the genotyping results was controlled through the use of genomic DNA from human cell lines with known *STAT5B* wild-type (WT) or N642H mutant alleles; the WT sequence of *STAT5B* was controlled for using Karpas 384 cells, and homo- or heterozygous genotypes were represented by the DERL-7 and DERL-2 cell lines, respectively. To determine the sensitivity of the dPCR assay targeting $STAT5B^{N642H}$, genomic DNA from Karpas 384 (WT *STAT5B*) and DERL-7 (homozygous $STAT5B^{N642H}$) cells was normalized to the same concentration (2,620 copies/µl) by measuring the concentration of single copy gene *RPP30* by dPCR. Karpas 384 cell DNA was then spiked with 0%, 1%, 5% or 10% DNA from DERL-7 cells, and $STAT5B^{N642H}$ and *RPP30* copy numbers were quantified using dPCR. $STAT5B^{N642H}$ variant allele frequency (VAF) was then calculated from the measured $STAT5B^{N642H}$ copy numbers as a percentage of *RPP30* copy numbers from each sample.

Statistics

GraphPad Prism v10.6.1 software was used for statistical analyses, applying unpaired two-tailed Student's t-tests for comparison of two groups, and two-way ANOVA with Tukey post-test for comparison of multiple groups with a time variable. $^{*}p < 0.05$, $^{**}p < 0.01$, $^{***}p < 0.001$, $^{****}p < 0.0001$.

Graphical licences

The cell line generation schematic was created using BioRender.com, under the agreement number KJ26MEJQCR.

Supplementary Tables

Supplementary Table 1: HSTCL Patient Samples

Patient #	Age	Sex	Tissue (FFPE)	Status of sample (FFPE)	Tissue (cryopreserved, viable)	Status of sample (cryopreserved, viable)	Tumor cell % (cryopreserved samples)	Healthy cell marker	Tumor cell markers
1	37	M	Bone marrow	Primary diagnosis	Peripheral blood	Relapse (splenectomy-SMILE-bortezomib)	15%	$CD5^+$	$CD3^+$ $CD56^+$ $TCR\gamma\delta^+$
2	75	F	Spleen	Primary diagnosis	Spleen	Primary diagnosis	5-10%	$CD20^+$	$CD2^+$ $CD3^+$ $CD7^+$
3	32	M	Spleen	Primary diagnosis, undergoing SMILE protocol (+21d)	Bone marrow	Relapse (splenectomy-SMILE-cyclophosphamide+ mitoxantrone+prednisolone +alemtuzumab)	45%	$CD20^+$	$CD2^+$ $CD3^+$ $CD7^+$
4	30	M	Spleen	Primary diagnosis	Spleen	Primary diagnosis	15%	$CD20^+$	$CD2^+$ $CD56^+$
5	50	M	Spleen	Primary diagnosis	Spleen	Primary diagnosis	5-10%	$CD20^+$	$CD2^+$ $CD3^+$ $CD7^+$

Supplementary Table 2: Western Blot Antibodies

Antibody	Company	Cat. #	Dilution
DYKDDDDK FLAG Tag (L5)	Biolegend	637302	1:500
pY-STAT5	Cell Signaling Technology	9314S/9351S	1:1000
STAT5	BD Biosciences	610191	1:1000
α-Tubulin	Santa Cruz Biotechnology	sc-32293	1:5000
Actin (C11)	Santa Cruz Biotechnology	sc-1615R	1:10000
β-actin (C4)	Santa Cruz Biotechnology	sc-47778	1:5000
HSC70	Santa Cruz Biotechnology	sc-7298	1:5000
IRDye 680RD Goat anti-Mouse IgG	LI-COR	926-68070	1:10000
IRDye 800CW Goat anti-Mouse IgG	LI-COR	926-32210	1:10000
IRDye 680RD Goat anti-Rabbit IgG	LI-COR	925-68071	1:10000
IRDye 800CW Goat anti-Rabbit IgG	LI-COR	926-32211	1:10000
IRDye 800CW Goat anti-Rat IgG	LI-COR	926-32219	1:10000

Supplementary Table 3: CRISPR-Cas9 Guide RNAs

Target	Forward sequence	Reverse sequence
h*STAT5B*_1	CACCGTAACGCTTGCATCTGATGAA	AAACTTCATCAGATGCAAGCGTTAC
h*STAT5B*_2	CACCGCCTCAAACGTCTGGTTGATC	AAACGATCAACCAGACGTTTGAGGC
non-targeting control_1	CACCGCGCTTCCGCGGCCCGTTCAA	AAACTTGAACGGGCCGCGGAAGCGC
non-targeting control_2	CACCGATCGTTTCCGCTTAAGGCG	AAACCGCCGTTAAGCGGAAACGATC

Supplementary Table 4: Primers

PCR Primers

Target	Forward sequence	Reverse sequence
STAT5B	GGCAATGGTTTGACGGTG	GGATCCACTGACTGTCCATT

Sanger Sequencing Primer

Target	Sequence
STAT5B	GCCTCATTGGAATGATGG

qRT-PCR Primers

Target	Forward sequence	Reverse sequence
STAT5B	GATCAAGCAAGTGGTCCC	CCAGATCGAAGTCCCCATCGG
GAPDH	CAAGGTCATCCATGACAACTTTG	GTCCACCACCCTGTTGCTGTAG

Supplementary Table 5: Flow Cytometry Antibodies

Surface staining - mouse				
**Target**	**Fluorochrome**	**Clone**	**Company**	**Cat. #**
CD90.2 (Thy1.2)	APC	53-2.1	Thermo Fisher Scientific	17-0902-81
CD90.2 (Thy1.2)	PE	53-2.1	Thermo Fisher Scientific	12-0902-82
CD3ε	FITC	145-2C11	Biolegend	100306
CD5	FITC	53-7.3	Biolegend	100605
CD4	PE	GK1.5	Thermo Fisher Scientific	12-0041-83
CD8α	PerCP-Cy5.5	53-6.7	Thermo Fisher Scientific	45-0081-82
CD11b	PerCP-Cy5.5	M1/70	Thermo Fisher Scientific	45-0112-80
NK1.1	PE/Cy7	PK136	Biolegend	108713
CD45.1 (Ly5.1)	BV650	A20	Biolegend	110735
CD45.1 (Ly5.1)	PE	A20	Thermo Fisher Scientific	12-0453-82
CD45.2 (Ly5.2)	PE/Cy5	104	BD Biosciences	552950
CD45.2 (Ly5.2)	BV605	104	Biolegend	109841
TCRβ	PE/Cy7	H57-597	BD Biosciences	560729

TCRδ	BV421	GL3	BD Biosciences	562892
TCRδ	FITC	GL3	Thermo Fisher Scientific	11-5711-85
Ter119	APC/Cy7	TER-119	Biolegend	116223
Rat IgG2a,κ	FITC	RTK2758	Biolegend	400505
Armenian hamster IgG	APC	HTK888	Biolegend	400911
Rat IgG2a,κ	PE	eBR2a	Thermo Fisher Scientific	12-4321-81
Rat IgG2b,κ	PE	RTK4530	Biolegend	400607
Armenian hamster IgG	FITC	HTK888	Biolegend	400905
Rat IgG2a,κ	PerCP-Cy5.5	eBR2a	Thermo Fisher Scientific	45-4321-80
Armenian hamster IgG	PE/Cy7	HTK888	Biolegend	400921
Armenian hamster IgG	BV421	HTK888	Biolegend	400935
**Surface staining - human**				
CD3ε	FITC	UCHT1	Biolegend	300405
CD3ε	APC/Cy7	HIT3a	Biolegend	300318
TCRγδ	PE	11F2	BD Biosciences	333141
TCRγδ	PE	B1	Biolegend	331210
TCRαβ	APC	IP26	Biolegend	306718
CD2	PE	TS1/8	Biolegend	309208
CD2	PE	S5.2	Biolegend	347405
CD2	FITC	RPA-2.10	Biolegend	300206
CD5	FITC	UCHT2	BD Biosciences	561896
CD5	PE/Cy7	L17F12	Biolegend	364007
CD4	PE	OKT4	Biolegend	317410
CD8	PE	HIT8a	Biolegend	300908
CD11b	APC	M1/70	Biolegend	101212
CD7	FITC	CD7-6B7	Biolegend	343104
CD56	APC	NCAM16.2	BD Biosciences	341027
CD56	FITC	HCD56	Biolegend	318304

CD20	PE/Cy7	2H7	Biolegend	302312
Rat IgG1, κ	APC	RTK2071	Biolegend	400411
Mouse IgG1, κ	FITC	MOPC-21	Biolegend	400107
Mouse IgG2b, κ	PE	MG2b-57	Biolegend	401208
**Intracellular staining - mouse**				
**Target**	**Fluorochrome**	**Clone**	**Company**	**Cat. #**
DYKDDDDK Tag (FLAG)	APC	L5	Biolegend	637307
CD3ε	eFluor450	145-2C11	Thermo Fisher Scientific	48-0031-82
TCRδ	FITC	GL3	Thermo Fisher Scientific	11-5711-85
TCRβ	APC	H57-597	BD Biosciences	553174
TIA-1	-	G-3	Santa Cruz Biotechnology	sc-166247
Goat anti-Mouse IgG Secondary Antibody	AlexaFluor647	Polyclonal	Thermo Fisher Scientific	A-21235
Armenian hamster IgG	FITC	HTK888	Biolegend	400905
**Intracellular staining - human**				
CD3ε	FITC	UCHT1	Biolegend	300405
TCRγδ	PE	11F2	BD Biosciences	333141
TCRαβ	APC	IP26	Biolegend	306718
TIA-1	-	G-3	Santa Cruz Biotechnology	sc-166247
Goat anti-Mouse IgG Secondary Antibody	AlexaFluor647	Polyclonal	Thermo Fisher Scientific	A-21235
Rat IgG1, κ	APC	RTK2071	Biolegend	400411
Mouse IgG2b, κ	PE	MG2b-57	Biolegend	401208
**Miscellaneous**				
**Target**	**Fluorochrome**	**Clone**	**Company**	**Cat. #**
Viability dye	eFluor780	-	Thermo Fisher Scientific	65-0865-14
Fc block	-	93	Biolegend	101302
Fc block	-	-	Biolegend	422301
DAPI	-	-	Biolegend	422801

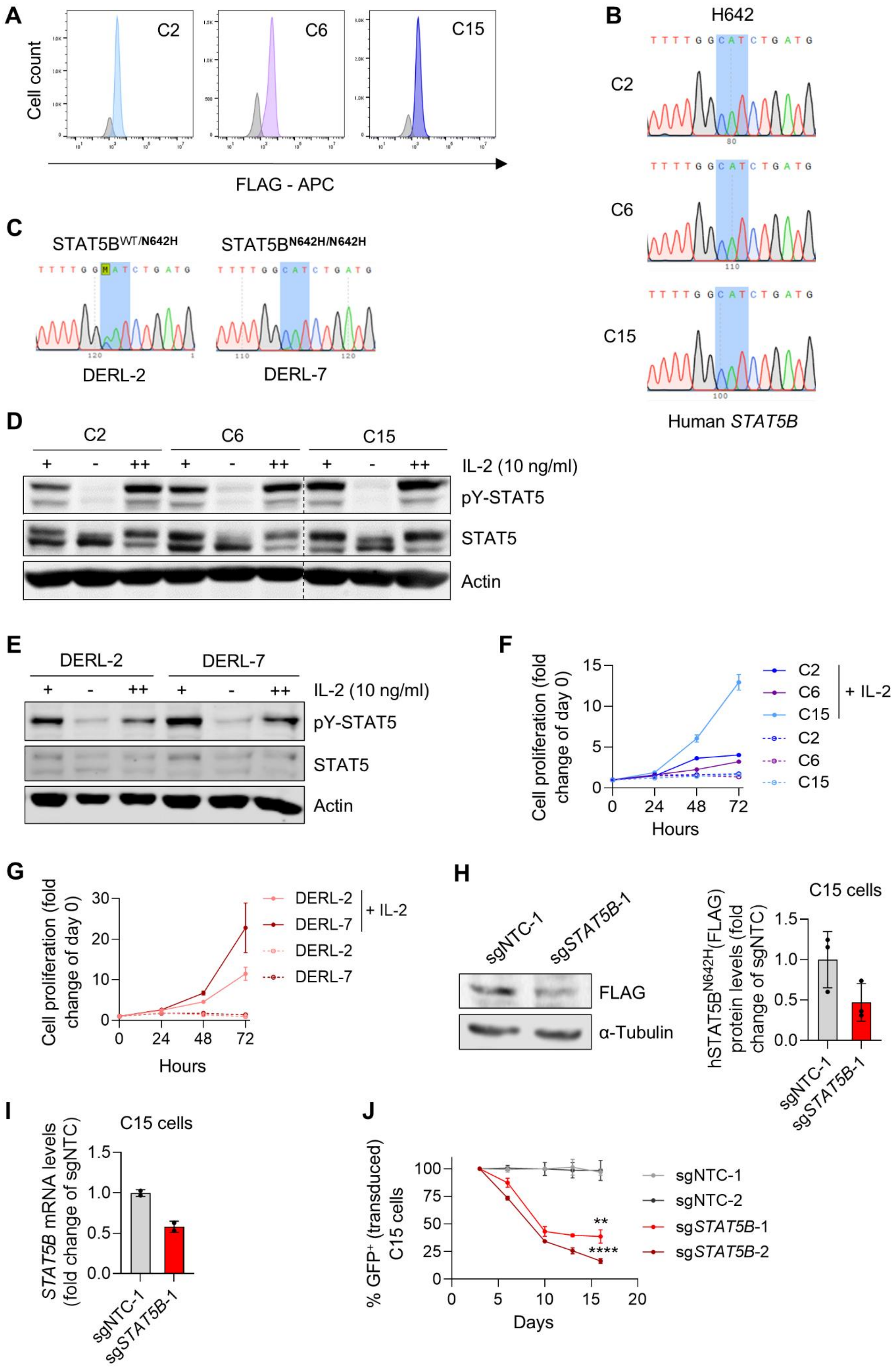
A
Cell count
C2
C6
C15
FLAG - APC
B
H642
C2
C6
C15
Human STAT5B
C
STAT5B WT/N642H
STAT5B N642H/N642H
DERL-2
DERL-7
D
C2
C6
C15
IL-2 (10 ng/ml)
pY-STAT5
STAT5
Actin
E
DERL-2
DERL-7
IL-2 (10 ng/ml)
pY-STAT5
STAT5
Actin
F
Cell proliferation (fold change of day 0)
Hours
+ IL-2
G
Cell proliferation (fold change of day 0)
Hours
DERL-2
DERL-7
+ IL-2
H
sgNTC-1
sgSTAT5B-1
FLAG
α-Tubulin
C15 cells
hSTAT5B N642H (FLAG) protein levels (fold change of sgNTC)
I
C15 cells
STAT5B mRNA levels (fold change of sgNTC)
J
% GFP+ (transduced) C15 cells
Days
sgNTC-1
sgNTC-2
sgSTAT5B-1
sgSTAT5B-2

Supplementary Figure 1. A) Representative histograms depicting mean fluorescence intensity of intracellular FLAG (hSTAT5B^{N642H} transgene) protein levels in C2, C6 and C15 γδTCL cells (colored peaks) compared with isotype control antibodies (grey peaks), as determined by flow cytometry (*n* = 3). **B-C)** Sanger sequencing of the SH2 domain of human *STAT5B* using genomic DNA isolated from B) C2, C6 and C15 murine cell lines, or C) DERL-2 and DERL-7 human HSTCL cell lines. The codon for amino acid position 642 is highlighted in blue. **D-E)** Western blots showing STAT5 activity in D) C2, C6 and C15 murine cell lines or E) DERL-2 and DERL-7 human cell lines cultured in media supplemented with IL-2 for 24 hr (+), starved of IL-2 for 8 hr (-), and then restimulated with IL-2 for 1 hr (++). Immunoblotting for pY-STAT5 and total STAT5 was performed, with actin serving as a loading control (representative blots shown; murine cell lines, *n* = 3; human cell lines, *n* = 2). Dashed lines indicate where lanes from the same immunoblots have been spliced together to aid interpretation. **F-G)** Cell proliferation of F) C2, C6 and C15 murine cell lines or G) DERL-2 and DERL-7 human cell lines in the presence or absence of IL-2 over 72 hr, measured by flow cytometry. Data are graphed as mean (± SD) of technical triplicates from one experiment, representative of two (human lines) or three (murine lines) independent experiments (*n* = 2-3). **H)** Western blot assessing STAT5B^{N642H}(FLAG) protein levels in C15 cells 4 or 11 days post CRISPR-Cas9 gene editing using a human *STAT5B*-targeting sgRNA or non-targeting control (NTC) sgRNA. Immunoblotting was performed using anti-FLAG-tag antibodies, with α-Tubulin serving as a loading control. A representative blot is shown (*left*) of three independent experiments. Band intensities were quantified, normalized to loading control and graphed as mean (± SD) (*right*; *n* = 3). **I)** Relative *STAT5B* mRNA levels in C15 cells 4 days post CRISPR-Cas9 gene editing using a human *STAT5B*-targeting sgRNA or NTC sgRNA. Gene expression was determined by qRT-PCR ($2^{-\Delta\Delta Ct}$ method, normalized to *GAPDH*). Data are graphed as mean (± SD) from two independent experiments (*n* = 2). **J)** Cell competition assay of C15 cells transduced with human *STAT5B*-targeting sgRNAs or NTC sgRNAs, both containing GFP markers, for CRISPR-Cas9 gene editing. Percentages of GFP+ cells were measured over time using flow cytometry. Data are graphed as mean (± SD) of technical triplicates from one

experiment, representative of three independent experiments (n = 3). **p < 0.01, ****p < 0.0001 (compared with sgNTC-1); two-way ANOVA with Tukey post-test for multiple comparisons.

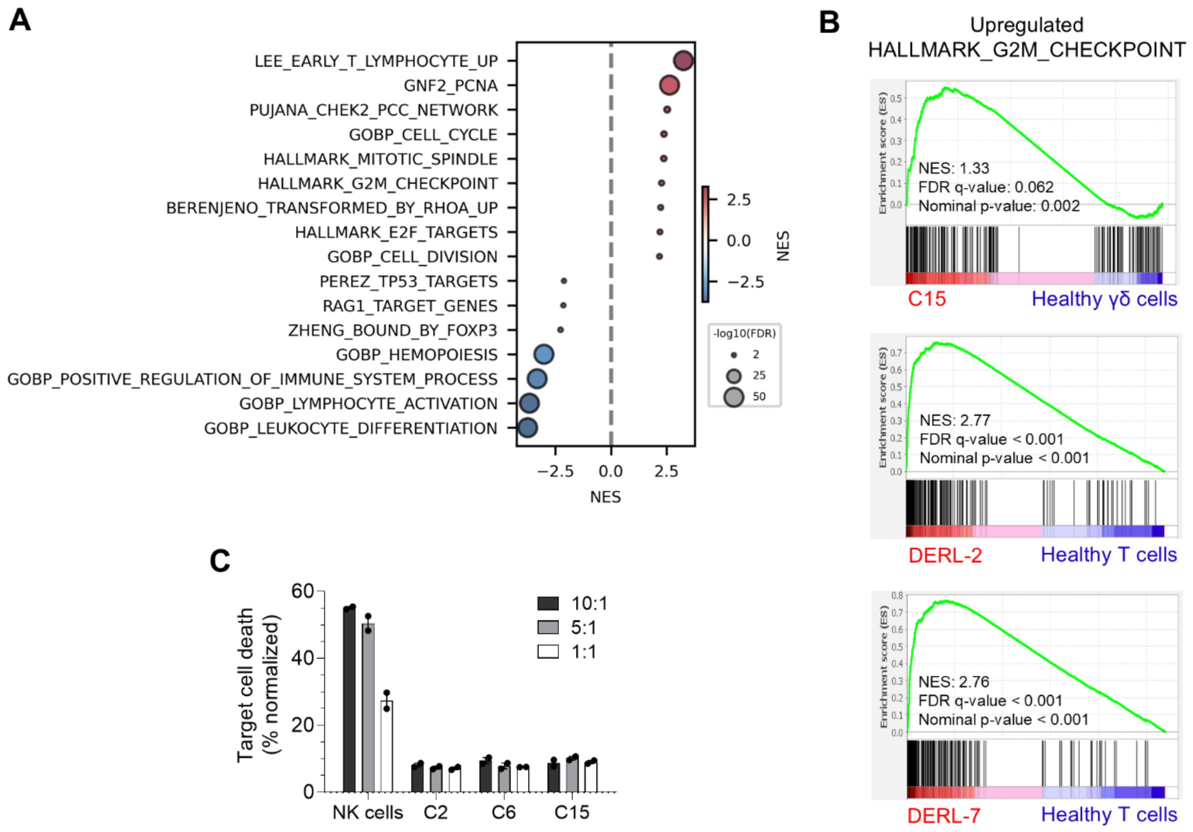

Supplementary Figure 2. A) Gene set enrichment analysis (GSEA) of overlapping differentially expressed genes (DEGs) across the murine and human cell lines and primary HSTCL patient samples, pre-ranked by log2 fold change, displayed as a bubble plot showing significantly up- (red) and down-regulated (blue) biological pathways; thresholds: |NES| > 2, FDR $q < 0.05$, nominal $p < 0.01$. Bubble size represents -log10 (FDR). NES, normalized enrichment score. **B)** GSEA of DEGs from the mouse C15, and human DERL-2 and DERL-7 cell lines, compared to the respective controls, displaying significant upregulation of G2/M checkpoint pathway genes. **C)** Cell based killing assay examining the cytotoxic capacity of C2, C6 and C15 cells against the target tumor cell line, YAC-1. Cells were seeded at 10:1, 5:1 and 1:1 ratios of γδTCL cells:target cells and incubated for 4 hr. Freshly isolated and IL-2 stimulated primary murine NK cells were used as a positive control. Target cell viability was measured by flow cytometry, and data are graphed as mean (± range) of technical duplicates, representative of three independent experiments ($n = 3$).

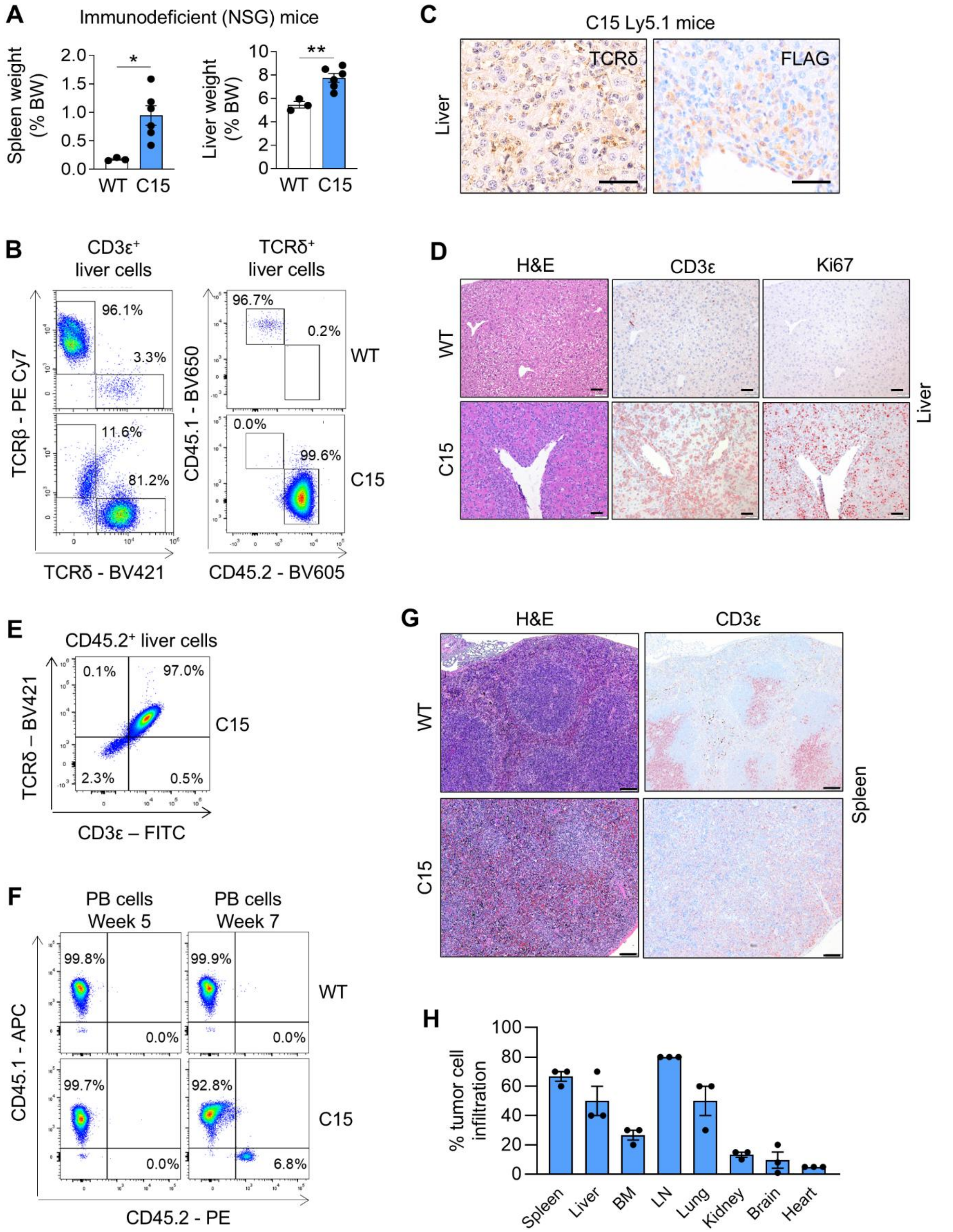

Supplementary Figure 3. A) Spleen and liver weights (as % of body weight, BW) of WT and diseased C15-recipient NSG mice. Data are graphed as mean (± SEM). **p* < 0.05, ***p* < 0.01; unpaired two-tailed Student's t-test. **B)** Representative FACS plots showing percentages of TCRβ^{+} and TCRδ^{+} liver cells (gated on CD3^{+} cells), and CD45.1^{+} and CD45.2^{+} liver cells (gated

on TCRδ$^+$ cells), from WT and C15-recipient Ly5.1 mice. **C)** Representative images from IHC analysis of TCRδ and FLAG staining in liver from C15-recipient Ly5.1 mice, imaged by light microscopy (scale bar = 50 μm). **D)** Representative images from IHC analysis of consecutive liver sections from WT or C15-recipient Ly5.1 mice, stained for CD3, Ki67 and H&E and imaged by light microscopy at 20x magnification (scale bar = 50 μm). **E)** Representative FACS plot showing percentages of CD3ε and TCRδ cell surface expression on CD45.2$^+$ liver cells from C15-recipient Ly5.1 mice. **F)** Representative FACS plots showing percentages of CD45.1$^+$ and CD45.2$^+$ cells in the peripheral blood (PB) of WT or C15-recipient Ly5.1 mice at 5- and 7-weeks post transplantation. **G)** Representative images of spleen morphology of WT or C15-recipient Ly5.1 mice using IHC stained for CD3 and H&E, and imaged by light microscopy at 10x magnification (scale bar = 100 μm). **H)** Quantification of tumor cell infiltration (%) into various organs of C15-recipient Ly5.1 mice (*n* = 3), using representative IHC images of CD3 staining.

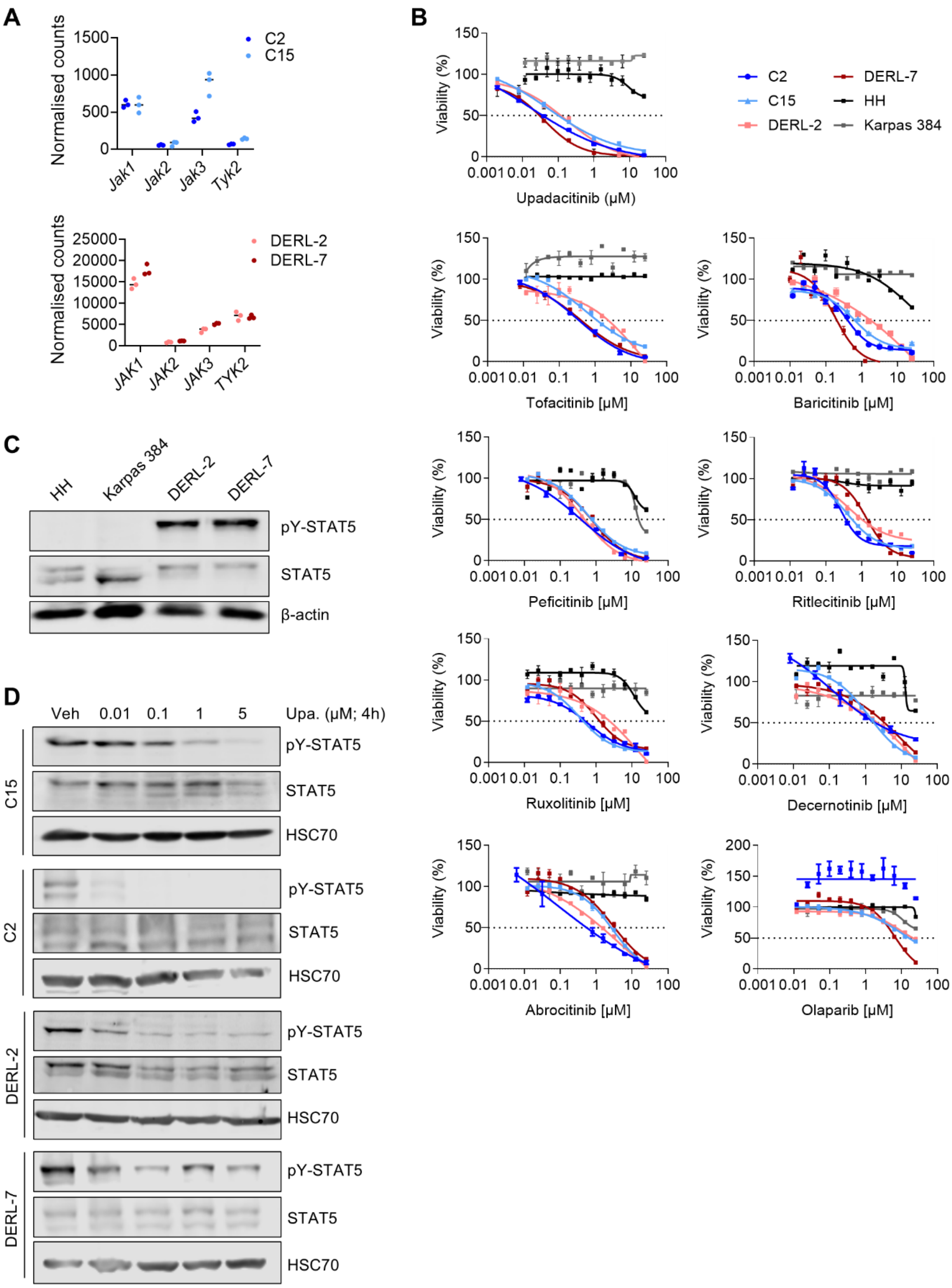

Supplementary Figure 4. A) Normalised counts of *Jak1, Jak2, Jak3* and *Tyk2* mRNA in murine C2 and C15 cell lines, and of *JAK1, JAK2, JAK3* and *TYK2* mRNA in human DERL-2 and DERL-7 cell lines, determined by RNA-seq (*n* = 3). **B)** Cell viability curves upon 48 hr treatment of the indicated drugs at various concentrations using murine (C2, C15) and human

(DERL-2, DERL-7) HSTCL cell lines, and control human (HH, Karpas 384) TCL cell lines. Data are graphed as mean (± SD) of technical triplicates from one experiment, representative of three independent experiments (*n* = 3). **C)** Western blot showing STAT5 activity in human TCL cell lines (HH, Karpas 384) and HSTCL cell lines (DERL-2, DERL-7). Immunoblotting for pY-STAT5 and total STAT5 was performed, with β-actin serving as a loading control (blots representative of two independent experiments; *n* = 2). **D)** Western blots showing STAT5 activity in C15, C2, DERL-2 and DERL-7 cells treated with upadacitinib for 4 hr at the indicated concentrations. During this time, cells were starved of IL-2 for 3.5 hr and then restimulated with 10 ng/mL IL-2 for 30 min. Immunoblotting for pY-STAT5 and total STAT5 was performed, with HSC70 serving as a loading control (blots representative of two independent experiments each; *n* = 2). Veh, vehicle (DMSO).

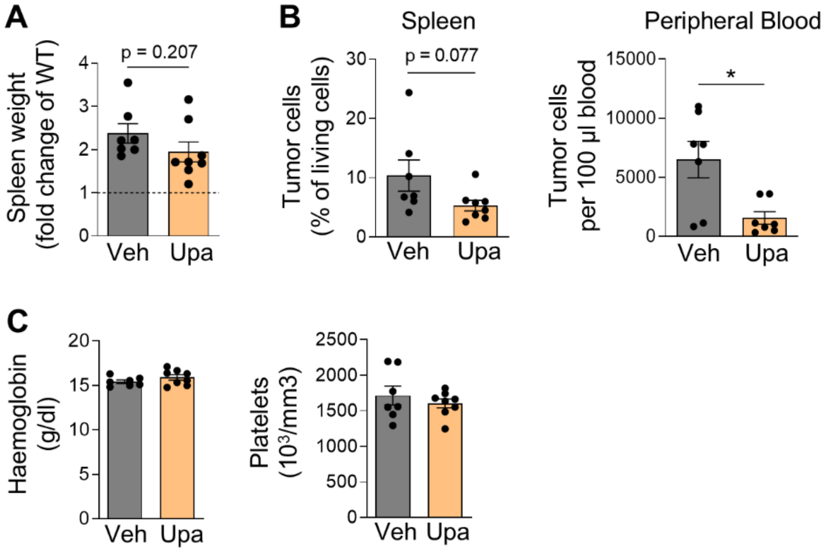

Supplementary Figure 5. A) Spleen weights of C15-recipient NSG mice treated with vehicle (veh; *n* = 7) or upadacitinib (upa; *n* = 8), graphed as fold change of the average spleen weight of WT NSG mice (dashed line). All mice were analyzed 56 days post-transplant. Data are graphed as mean (± SEM); *p* value determined using an unpaired two-tailed Student's t-test. **B)** Quantification of intracellular $FLAG^+$ tumor cell numbers in the spleen and peripheral blood of vehicle or upadacitinib treated C15-recipient NSG mice, analyzed by flow cytometry. Data are graphed as mean (± SEM). *$p < 0.05$; unpaired two-tailed Student's t-test. **C)** Haemoglobin and platelet levels in whole blood of vehicle (*n* = 7) or upadacitinib (*n* = 8) treated C15-recipient NSG mice, analyzed with an animal blood counter (Scil Vet ABC). Data are graphed as mean (± SEM).

A

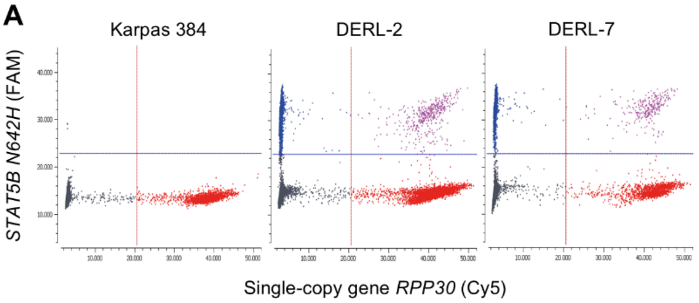

Cell line	*STAT5B*^{N642H} (cp/µl)	*RPP30* (cp/µl)	*STAT5B*^{N642H} VAF (%)
Karpas 384	5 ± 4	2189 ± 100	0.2 ± 0.2
DERL-2	2330 ± 96	4259 ± 133	54.7 ± 2.3
DERL-7	3381 ± 147	3119 ± 141	108.4 ± 4.7

B

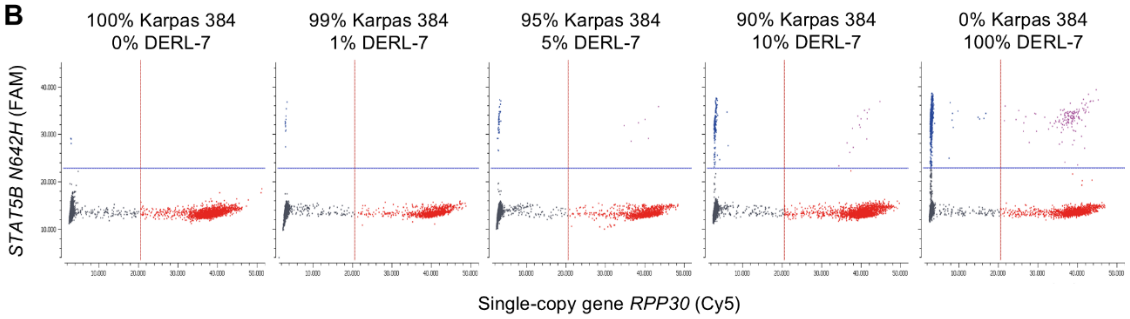

C

% DERL-7 DNA	*STAT5B*^{N642H} (cp/µl)	*RPP30* (cp/µl)	*STAT5B*^{N642H} VAF (%)
0	4.5 ± 4.4	2188.8 ± 99.6	0.2 ± 0.2
1	20.9 ± 11.4	2170.0 ± 118.9	1.0 ± 0.5
5	99.5 ± 27.6	2186.3 ± 132.7	4.6 ± 1.3
10	197.5 ± 27.3	2013.8 ± 88.8	9.8 ± 1.4
100	2032.5 ± 92.9	1788.8 ± 86.8	113.6 ± 5.2

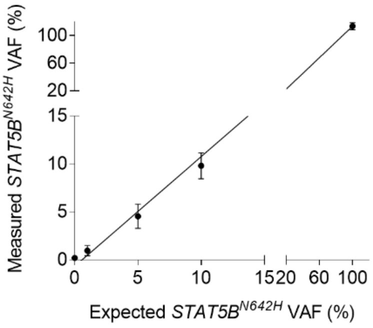

D

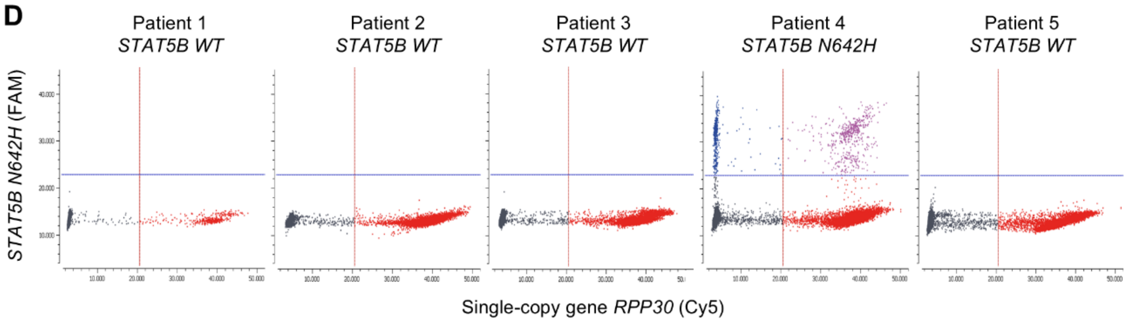

Supplementary Figure 6. A-C) Digital PCR (dPCR) variant-specific assay validation for the detection of the *STAT5B*^{N642H} SNP. **A)** Variant allele frequency (VAF) of *STAT5B*^{N642H} measured by dPCR using genomic DNA from Karpas 384 (WT *STAT5B*), DERL-2 (heterozygous *STAT5B*^{N642H}), and DERL-7 (homozygous *STAT5B*^{N642H}) cells. (*Left*) Data are presented as two-dimensional dPCR scatter plots, with each panel representing a separate measurement of reaction mix containing DNA partitioned into droplets and assessed for

STAT5B^{N642H} and *RPP30*. The arbitrary threshold distinguishing positive droplets from the background of negative droplets is indicated by a blue (FAM fluorophore) or a red (Cy5 fluorophore) line. (*Right*) Table displaying dPCR data with quantifications of *STAT5B*^{N642H} and *RPP30* copy numbers, and calculation of *STAT5B*^{N642H} variant allele frequency (VAF). Data are presented as mean ± Poisson 95% confidence intervals (as determined by the dPCR software). **B-C)** Measurement of the sensitivity of the dPCR variant-specific assay, using genomic DNA from Karpas 384 cells (WT *STAT5B*) spiked with 0, 1, 5 or 10% DNA from DERL-7 cells (homozygous *STAT5B*^{N642H}). *STAT5B*^{N642H} and *RPP30* copy numbers were measured by dPCR. Two-dimensional dPCR scatter plots are shown (B), dPCR data measurements and quantifications of *STAT5B*^{N642H} VAF as percentage of *RPP30* copy number are listed (C, *left*), and the measured *STAT5B*^{N642H} VAFs are graphed against the expected VAFs (C, *right*). Data are presented as mean ± Poisson 95% confidence intervals (as determined by the dPCR software). **D)** Screening for the *STAT5B*^{N642H} variant by dPCR using genomic DNA isolated from FFPE tumor tissues from five HSTCL patients. Two-dimensional dPCR scatter plots are shown, measuring *STAT5B*^{N642H} and *RPP30* copy numbers.

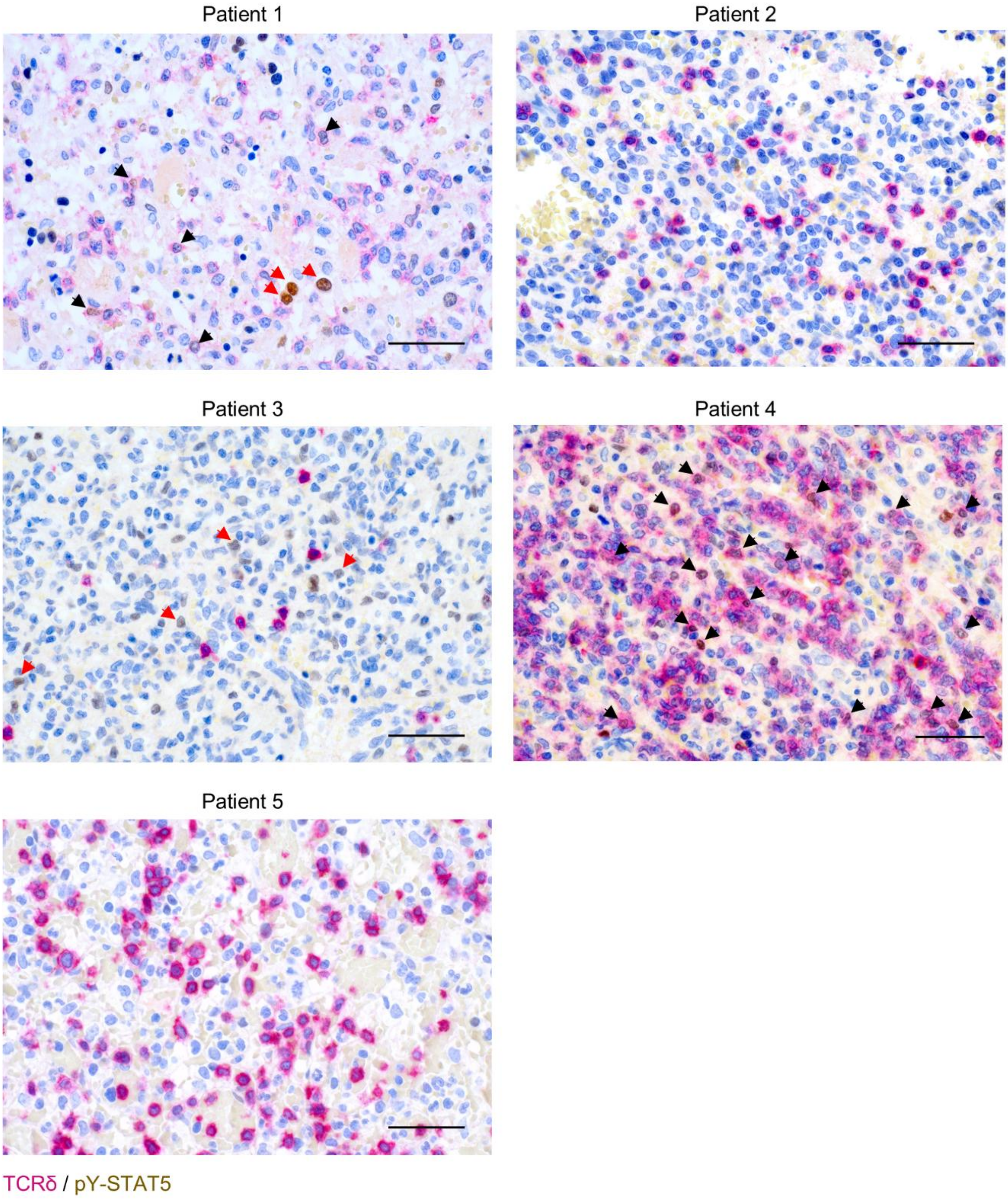

Supplementary Figure 7. Representative images from IHC analysis of TCRδ (magenta) and pY-STAT5 (brown) double staining in the bone marrow (patient 1) or spleen (patients 2-5) of HSTCL patients, imaged by light microscopy (scale bar = 50 µm). Black arrows indicate tumor cells with TCRδ and pY-STAT5 double staining; red arrows indicate positive pY-STAT5 staining in surrounding, non-malignant cells (e.g. strong nuclear staining in erythroid precursors, patient 1; weak nuclear staining in reactive lymphoid cells; patient 3).

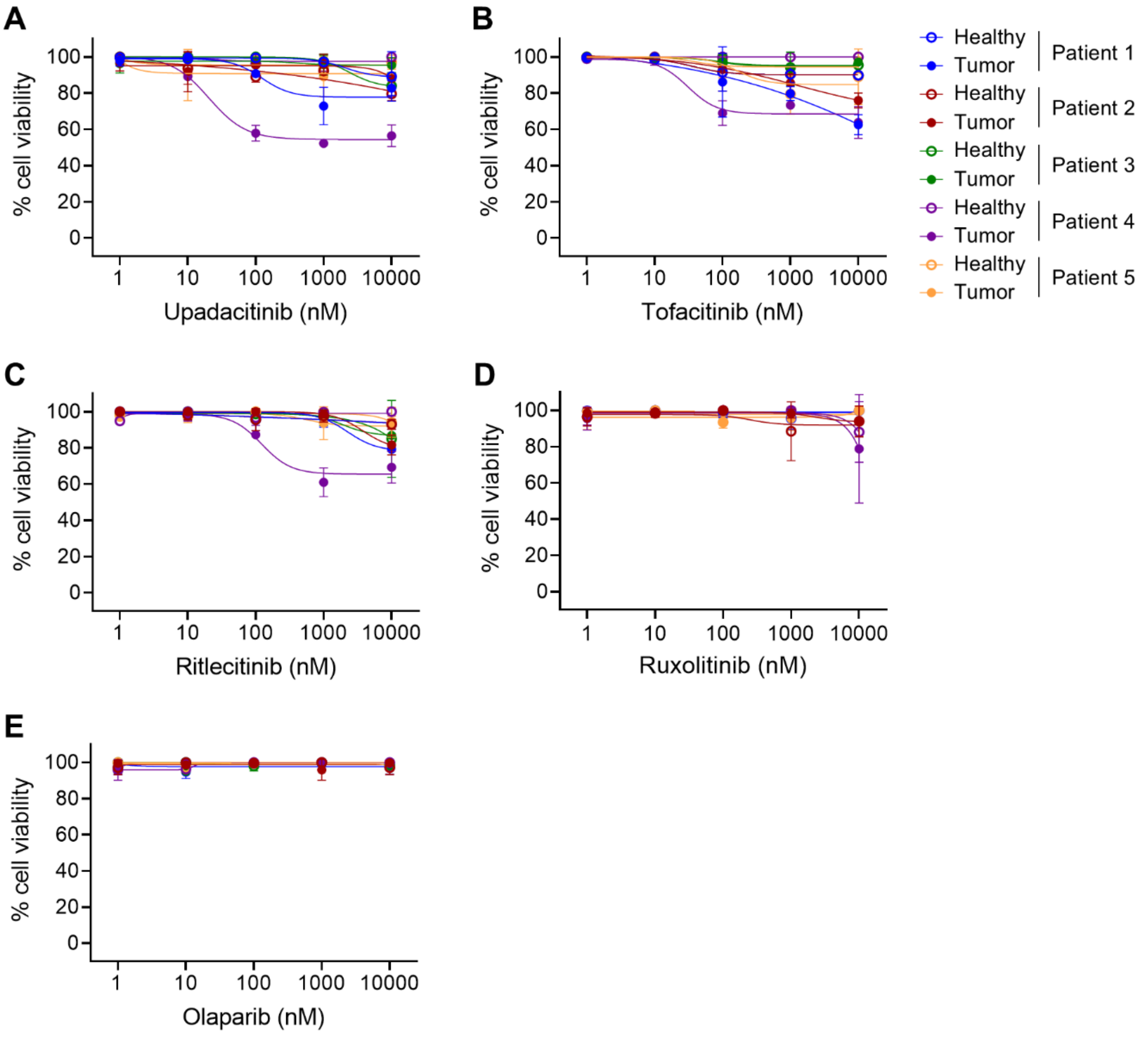

Supplementary Figure 8. A-E) Cell viability curves upon 24 hr treatment of A) upadacitinib, B) tofacitinib, C) ritlecitinib, D) ruxolitinib or E) olaparib at various concentrations on primary HSTCL patient samples containing both tumor and healthy cell populations, distinguished by cell surface markers (see Table S1) and flow cytometry. Data are graphed as mean (± SD) of technical duplicates from one experiment (*n* = 1).

Supplementary References

1. Pham HTT, Maurer B, Prchal-Murphy M, et al. STAT5BN642H is a driver mutation for T cell neoplasia. *J Clin Invest*. 2018;128(1):387-401.
2. de Araujo ED, Erdogan F, Neubauer HA, et al. Structural and functional consequences of the STAT5B(N642H) driver mutation. *Nat Commun*. 2019;10(1):2517.
3. Picelli S, Faridani OR, Bjorklund AK, Winberg G, Sagasser S, Sandberg R. Full-length RNA-seq from single cells using Smart-seq2. *Nat Protoc*. 2014;9(1):171-181.
4. Schmieder R, Edwards R. Quality control and preprocessing of metagenomic datasets. *Bioinformatics*. 2011;27(6):863-864.
5. Dobin A, Davis CA, Schlesinger F, et al. STAR: ultrafast universal RNA-seq aligner. *Bioinformatics*. 2013;29(1):15-21.
6. Li H, Handsaker B, Wysoker A, et al. The Sequence Alignment/Map format and SAMtools. *Bioinformatics*. 2009;25(16):2078-2079.
7. Danecek P, Bonfield JK, Liddle J, et al. Twelve years of SAMtools and BCFtools. *Gigascience*. 2021;10(2):giab008.
8. Liao Y, Smyth GK, Shi W. featureCounts: an efficient general purpose program for assigning sequence reads to genomic features. *Bioinformatics*. 2014;30(7):923-930.
9. Love MI, Huber W, Anders S. Moderated estimation of fold change and dispersion for RNA-seq data with DESeq2. *Genome Biol*. 2014;15(12):550.
10. Wickham H. Ggplot2: Elegant Graphics for Data Analysis (ed 2nd). New York: Springer; 2016.
11. Carlson M. Orthology.Eg.Db: Orthology Mapping Package; 2024.
12. Carlson M. Org.Mm.Eg.Db: Genome Wide Annotation for Mouse; 2024.
13. Carlson M. Org.Hs.Eg.Db: Genome Wide Annotation for Human. 2024.
14. Finalet Ferreiro J, Rouhigharabaei L, Urbankova H, et al. Integrative genomic and transcriptomic analysis identified candidate genes implicated in the pathogenesis of hepatosplenic T-cell lymphoma. *PLoS One*. 2014;9(7):e102977.

15. Gu Z, Eils R, Schlesner M. Complex heatmaps reveal patterns and correlations in multidimensional genomic data. *Bioinformatics*. 2016;32(18):2847-2849.
16. Subramanian A, Tamayo P, Mootha VK, et al. Gene set enrichment analysis: a knowledge-based approach for interpreting genome-wide expression profiles. *Proc Natl Acad Sci U S A*. 2005;102(43):15545-15550.
17. Nabekura T, Gotthardt D, Niizuma K, et al. Cutting Edge: NKG2D Signaling Enhances NK Cell Responses but Alone Is Insufficient To Drive Expansion during Mouse Cytomegalovirus Infection. *J Immunol*. 2017;199(5):1567-1571.
18. Potdar S, Ianevski F, Ianevski A, et al. Breeze 2.0: an interactive web-tool for visual analysis and comparison of drug response data. *Nucleic Acids Res*. 2023;51(W1):W57-W61.
19. Suske T, Sorger H, Manhart G, et al. Hyperactive STAT5 hijacks T cell receptor signaling and drives immature T cell acute lymphoblastic leukemia. *J Clin Invest*. 2024;134(8):e168536.
20. Kazianka L, Pichler A, Agreiter C, et al. Comparing functional and genomic-based precision medicine in blood cancer patients. *Hemasphere*. 2025;9(4):e70129.
21. Ye J, Coulouris G, Zaretskaya I, Cutcutache I, Rozen S, Madden TL. Primer-BLAST: A tool to design target-specific primers for polymerase chain reaction. *BMC Bioinformatics*. 2012;13:134.
22. Malkki M, Petersdorf EW. Genotyping of single nucleotide polymorphisms by 5′ nuclease allelic discrimination. *Methods in Molecular Biology*. 2012;882:173-182.
23. Echwald SM, Andreasen D, Mouritzen P. LNA™ Adding New Functionality to PCR. PCR Technology: Current Innovations, Third Edition; 2013:87-102.
24. Wen TT, Zhang XH, Lippuner C, Schiff M, Stuber F. Development and Evaluation of a Droplet Digital PCR Assay for 8p23 β-Defensin Cluster Copy Number Determination. *Molecular Diagnosis & Therapy*. 2021;25(5):607-615.
